# Electrophilicity Modulation for Sub‐ppm Visualization and Discrimination of EDA

**DOI:** 10.1002/advs.202400361

**Published:** 2024-03-06

**Authors:** Hao Zhao, Yuan Liu, Gaosheng Li, Da Lei, Yuwan Du, Yudong Li, Hui Tang, Xincun Dou

**Affiliations:** ^1^ Key Laboratory of Xinjiang Phytomedicine Resource and Utilization Ministry of Education School of Pharmacy Shihezi University Shihezi 832000 China; ^2^ Xinjiang Key Laboratory of Trace Chemical Substances Sensing Xinjiang Technical Institute of Physics and Chemistry Chinese Academy of Sciences Urumqi 830011 China; ^3^ Center of Materials Science and Optoelectronics Engineering University of Chinese Academy of Sciences Beijing 100049 China; ^4^ Key Laboratory of Improvised Explosive Chemicals for State Market Regulation Urumqi 830011 China

**Keywords:** amines, CNN, nucleophilicity, optical sensing, trace detection

## Abstract

Precise and timely recognition of hazardous chemical substances is of great significance for safeguarding human health, ecological environment, public security, etc., especially crucial for adopting appropriate disposition measures. Up to now, there remains a practical challenge to sensitively detect and differentiate organic amines with similar chemical structures with intuitive analysis outcomes. Here, a unique optical probe with two electrophilic recognition sites for rapid and ultra‐sensitive ratiometric fluorescence detection of ethylenediamine (EDA) is presented, while producing distinct fluorescence signals to its structural analog. The probe exhibits ppb/nmol level sensitivity to liquidous and gaseous EDA, specific recognition toward EDA without disturbance to up to 28 potential interferents, as well as rapid fluorescence response within 0.2 s. By further combining the portable sensing chip with the convolutional algorithm endowed with image processing, this work cracked the problem of precisely discriminating the target and non‐targets at extremely low concentrations.

## Graphical abstract

A novel sensing strategy based on a unique differentiated‐nucleophilic mechanism is established with a boosted response to trace EDA either in liquid or gas and a distinguishable response for its structure analog—N_2_H_4_. This design concept is displayed as a telescope with tiny nucleophilicity discrimination ability toward the yachts labeled with EDA or N_2_H_4_ shown as blue emission or quenched fluorescence, respectively.

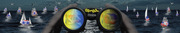

## 1 Introduction

Organic amines have a diverse range of applications,^[^ ^1^, ^2^ ^]^ especially as one of the major raw materials in manufacturing pharmaceuticals, pesticides, dyes, paints, etc.^[^ ^3^, ^4^ ^]^ For instance, ethylenediamine (EDA)^[^ ^5^ ^]^ and hydrazine^[^ ^6^ ^]^ act as a primary ingredient for producing the smooth muscle relaxant aminophylline and anti‐tuberculosis drugs methyldopa, while aniline and naphthylamine fall under the crucial raw materials category for preparing azo dye.^[^ ^7^ ^]^ Whereas, a portion of organic amines are listed as hazardous materials as their presence poses a severe threat to public security, human health, and environment sustainability due to their inherent toxicity, corrosivity, volatility, combustible and explosible properties.^[^ ^8^, ^9^, ^10^ ^]^ Among them, EDA is categorized as hazardous material because of its severe irritation and irreversible damage to the skin and nasal mucous, even aquatic creatures. Hence, both the World Health Organization (WHO)^[^ ^11^ ^]^ and the European Union^[^ ^12^ ^]^ have explicitly stipulated the occupational exposure thresholds for EDA atmosphere and solution as 10 ppm and 0.5 mg L^−1^ (8.3 µm), respectively, to guard against its potential menace during storage, application as well as discharge.^[^ ^13^, ^14^ ^]^ Thus, this arouses intense attention to developing sorts of laboratory instrument‐based and sensing technique‐based^[^ ^15^ ^]^ methodology to detect trace EDA, involving high‐performance liquid chromatography (HPLC),^[^ ^16^ ^]^ gas/liquid chromatography–mass spectrometry (GC/LC–MS),^[^ ^17^ ^]^ electrochemistry,^[^ ^18^ ^]^ fluorometry,^[^ ^19^, ^20^ ^]^ and colorimetry,^[^ ^21^, ^22^ ^]^ etc. Visualized optical sensing methods (e.g., fluorometry, colorimetry) with unique and well‐known merits have been widely applied in on‐site analyzing trace substances, accordingly, some optical sensing research has been reported for EDA detection upon the strong nucleophilicity of its primary amines.^[^ ^23^, ^24^, ^25^ ^]^ Although some studies have achieved a highly sensitive response toward trace EDA for catering to the specified thresholds,^[^ ^26^ ^]^ there still remains a challenge in differentiating EDA from other structurally similar analogs, especially for another hazardous chemical with highly similar structure and characteristics—hydrazine (N_2_H_4_), for which the purposely improper usage and accidental leakage also seriously threaten social stabilization and public health.^[^ ^27^, ^28^ ^]^ For example, the Schiff base mechanism was applied to develop the fluorescent probes (e.g., xanthene‐CHO probe, phenanthridine conjugated probe) for covalently interacting with EDA, yielding a detection limit as low as nm level, and being undisturbed to up to 22 interferents, while these probes exhibited the same fluorescent signals to EDA and hydrazine.^[^ ^29^, ^30^ ^]^ Even more, by applying the unique ring formation mechanism upon the presence of EDA, a sensing reagent composed of *o*‐phthalaldehyde and thiol could specifically detect EDA with the dual‐mode optical response, while the presence of hydrazine and other 28 interferents show the same non‐responsive phenomena.^[^ ^31^ ^]^ Hence, there remains significant demand for on‐site and reliably detecting EDA, meanwhile, outputting individually distinct sensing signals for more substances that hold similar hazardous characteristics (e.g., hydrazine), to achieve early and precise alarm as well as proper emergency measures.^[^ ^32^ ^]^

The core of the visualized optical probe lies in the recognition groups^[^ ^33^ ^]^ which covalently or non‐covalently interact with the analyte to bring about the visualized fluorescent and/or colorimetric signal upon the diverse photophysical mechanisms, such as photoinduced electron transfer (PET),^[^ ^34^ ^]^ intramolecular charge transfer (ICT),^[^ ^35^ ^]^ excited‐state intramolecular proton transfer (ESIPT),^[^ ^36^ ^]^ as well as aggregation‐induced emission (AIE),^[^ ^37^ ^]^ etc. Both EDA and hydrazine belong to the aliphatic primary amines, which are highly similar in structure and physical‐chemical properties, being particularly reflected in strong nucleophilicity.^[^ ^38^ ^]^ Most of the relevant optical probes were designed to contain an electrophilic group (e.g., aldehyde, ketone) by applying the Schiff base reaction to recognize the nucleophilic amine in EDA or hydrazine, thus, they normally yielded similar imine‐involved products with similar optical response signals.^[^ ^39^ ^]^ Upon parsing their structures in detail, it can be found that these two molecules carry different numbers of carbon atoms which lead to a tiny difference in chemical property, especially influencing the electron property on the amine groups.^[^ ^40^ ^]^ Therefore, there would be a chance to finely design the probe structure containing recognition sites with different activities to differentiate them and output distinguishable signals.^[^ ^41^ ^]^ For which there has been successful attempt by equipping two unsaturated double bonds with different activities within one probe to discriminate two oxides, KMnO_4_ and NaClO, according to their difference in oxidation ability.^[^ ^42^ ^]^ Moreover, whatever signal output modes (e.g., turn‐on, turn‐off, or ratiometry), the visualized responsive phenomenon is quite ambiguous for naked‐eye observation when the analyte is at the trace level.^[^ ^43^, ^44^ ^]^ Nowadays, leveraging on the rapid growth of deep learning techniques, typified by visual geometry group (VGG, e.g., VGG‐16), the convolutional neural network (CNN) has been paid much attention in image classification for tiny difference discrimination.^[^ ^45^, ^46^ ^]^ Hence, if one can propose a solution to develop a novel optical sensing strategy for differentiating EDA and other similar hazardous substances (e.g., hydrazine), and further apply algorithmic processing of images to lift the differentiating ability for the trace analytes.

Herein, based on a unique differentiated‐nucleophilic mechanism, a novel fluorescence sensing method was established with a boosted response to trace EDA either in liquid or gas and a distinguishable response for its highly similar structure analog—hydrazine. A benzothiazole probe was constructed by employing the electron‐deficient C═C as the recognition group to electrophilically react with EDA and decreasing the ICT extent of the probe at the excited state, inducing the ratiometric fluorescence response from yellow to blue. The sensing performances toward EDA were verified including nm (liquid) / ppb (vapor) level sensitivity, rapid response (≈0.2 s), and specific recognition without influence from 28 interferents. Especially, the precise recognition of EDA and hydrazine at low concentrations was realized by further incorporating CNN image processing. Furthermore, the accurate analysis of EDA in complicated samples has been verified regardless of the physical forms of the samples through a portable sensing chip, demonstrating the promising application potential in the storage and transportation of hazardous chemicals, industrial emissions, and pharmaceutical manufacture.

## 2 Results and Discussions

### 2.1 Theoretical Computation Analysis of Optical Sensing Mechanism

**Figure 1:**
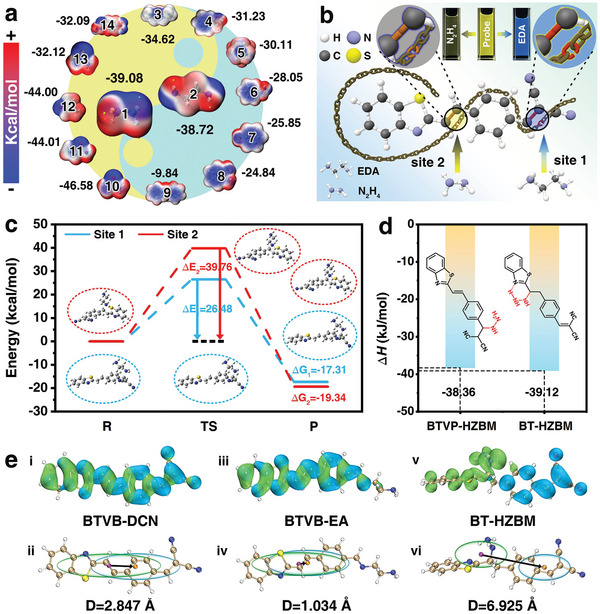
Theoretical computation analysis of the sensing mechanism of the BTVB‐DCN probe toward EDA. a) Electrostatic potential (ESP) distribution of the common organic amines which are listed as hazardous materials, 1) hydrazine (N_2_H_4_), 2) EDA, 3) diethylamine, 4) phenethylamine, 5) urotropine, 6) phenylhydrazine, 7) aniline, 8) *o*‐phenylenediamine, 9) diphenylamine, 10) urea, 11) acrylamide, 12) *N*,*N*‐dimethylformamide, 13) thiourea, 14) *p*‐nitrobenzohydrazide; b) Schematic representation of the nucleophilic recognition mechanisms between the probe toward EDA and hydrazine; c) Potential energy profiles for the reaction process between the BTVB‐DCN probe and EDA with zero‐point vibration corrected energies (kcal mol^−1^) relative to the reactants, the single point energy barrier (Δ*E*) and Gibbs free energy barrier (Δ*G*) were labeled; d) Thermodynamic enthalpy change (Δ*H*) for two hypothesized products after the BTVB‐DCN probe interacting with N_2_H_4_; e) Hole‐electron distribution analysis for the BTVB‐DCN probe and the products BTVB‐EA, BT‐HZBM: i,iii,v) the hole and electron distributions, ii,iv,vi) the *C*_hole_/*C*_electron_ plots smoothly transformed from the hole and electron distributions, the centroids of the *C*_hole_ and *C*_electron_ were marked by purple and orange spheres, respectively, and the charge transfer distances were labeled as D.

The systematic structural analyses (**Figure** **1**a) on the common hazardous organic amines show that EDA and hydrazine (N_2_H_4_) have stronger nucleophilicity around the amino group, and hydrazine shows a little bit more negative (in blue) electrostatic potential (ESP) values of −39.08 Kcal mol^−1^ than that of EDA (−38.72 Kcal mol^−1^). Thus, an optical probe (2‐(4‐(2‐(benzo[d]thiazol‐2‐yl)vinyl)benzylidene)malononitrile, BTVB‐DCN) was proposed with distinct responses to EDA and hydrazine (Figure 1b; Scheme S1, Figures S1–S9, Table S1, Supporting Information) by carrying two carbon‐carbon double bonds (C═C) with electron‐deficiency but in different degrees (shown as different positive ESP values, Figure S10, Supporting Information), where would be the most vulnerable regions for being nucleophilically attacked by the amino group. Experimentally, the specific recognition of the probe toward EDA and hydrazine with ratiometric and quenched fluorescent responses was proven (Figure S11, Supporting Information).

To gain a deep insight into the recognition process and the optical sensing response mechanism of the BTVB‐DCN probe toward EDA and hydrazine, a series of quantum chemistry computing studies were carried out upon the available computational platforms, e.g., Gaussian 09,^[^ ^47^ ^]^ Multiwfn software,^[^ ^48^ ^]^ VMD program.^[^ ^49^ ^]^ First of all, the single point energy barrier (Δ*E*) and the Gibbs free energy barriers (Δ*G*) of the reactions between EDA and two C═C sites of the probe were evaluated, for which Δ*E* for the reaction at site 1 (26.48 kcal mol^−1^) is less than that at site 2 (39.76 kcal mol^−1^) and Δ*G* values at two sites were similar, illustrating the recognition reaction at site 1 needs less energy to occur and generate similarly stable product (Figure 1c). Combining the structural characterization for the hypothesized product based on the high‐resolution mass spectrum (HRMS) and the infrared spectrum (IR) (Figures S12–S14, Supporting Information) with the above analyses, the sensing mechanism was explicated as the nucleophilic attack from the amino of EDA toward the C═C at site 1 of the probe. In terms of the sensing mechanism of the probe toward hydrazine, the peak of 346.1113 (m/z) (M+H^+^) in HRMS spectrum (Figure S15a, Supporting Information) collected after the probe interacted with hydrazine (N_2_H_4_) indicates that there were two potential reaction routes and corresponding products (Figure S15b, Supporting Information). Furthermore, the thermodynamic enthalpy change (Δ*H*) for the formation of these two theoretical products showed that the reaction for producing BT‐HZBM could release more heat, indicating the reaction between hydrazine and probe should be at site 2 with the more stable product of BT‐HZBM (Figure 1d).

Furthermore, the hole‐electron analysis of the BTVB‐DCN probe shows a relatively separated distribution while the holes are mainly distributed at the benzothiazole ring and the electrons are more concentrated around the dicyanovinyl of the probe (Figure 1e(i)). Comparably, the product BTVB‐EA after the probe interacting with EDA shows less separation of the hole‐electron distribution (Figure 1e(iii)),^[^ ^50^ ^]^ this distribution change complies well with the centroid distance (D) decrement of the hole (*C*_hole_) and the electron (*C*_electron_) before and after the probe reacting with EDA from 2.847 to 1.034 Å, suggesting an intramolecular charge transferring process (Figure 1e(ii,iv)).

Moreover, the fragment transition density matrixes (Figure S16, Supporting Information) upon the division of the probe and the product BTVB‐EA into four fragments show the distribution trend of the hole‐electron from separated distribution at fragments 1 (electron) and 4 (hole) to more concentrated distribution for both electron and hole at fragment 1, also agreeing well with the charge transferring process. The molecular orbital (MO) analysis (Table S2, Supporting Information) was further conducted for a better understanding of the optical response mechanism. It shows that the fluorescence emissions of the probe and the product were mainly contributed by the MO^82^ → MO^81^ transition with an increase of the energy gap from 2.932 eV (for the probe) to 3.497 eV (for the product) (Figure S16c, Supporting Information). This indicates a blue shift of the emissions for the probe and product, which aligns well with the experimental emissions from 560 to 450 nm (Figure S10b, Supporting Information) and the fitting emissions from 522 to 472 nm (Figure S16c, Supporting Information). The oscillator strength (*f*) values exhibited a decreased trend for the probe and the product from 2.170 to 1.876, which indicates a weakening fluorescence intensity entirely consistent with the experimental emission intensities. Regarding the distinctive response of the probe toward hydrazine (N_2_H_4_), similar analyses were carried out. From the hole‐electron analysis, the hypothesized product BTVP‐HZBM exhibited a smaller separation and the product BT‐HZBM had a larger separation compared to that of the probe with the D changes from 2.847 to 1.524, and 6.925 Å, respectively (Figure 1e(v,vi); Figure S17a,b, Supporting Information). The simulated fluorescence spectrum of the hypothesized product BTVP‐HZBM exhibited an emission at 452 nm with an *f* of 1.651, suggesting a blue‐shift compared to the simulated emission of the probe at 522 nm (Figures S16c(i) and S17c(i), Supporting Information), and the disagreement with the experimental quenched fluorescence after the probe interacting with hydrazine. While the product BT‐HZBM was found with a quenched emission in the simulated spectrum with an *f* of zero (Figure S17c(ii), Supporting Information), aligning well with the quenched fluorescent phenomenon, this also supports the correct product and sensing mechanism of the probe reacting with hydrazine at site 2. Hence, the proposed probe BTVB‐DCN possessed differentiated nucleophilic recognition mechanisms toward EDA and hydrazine, thus, yielding discriminable optical responses.

### 2.2 Fluorescent Sensing Performances of the BTVB‐DCN Probe to EDA Solution

**Figure 2:**
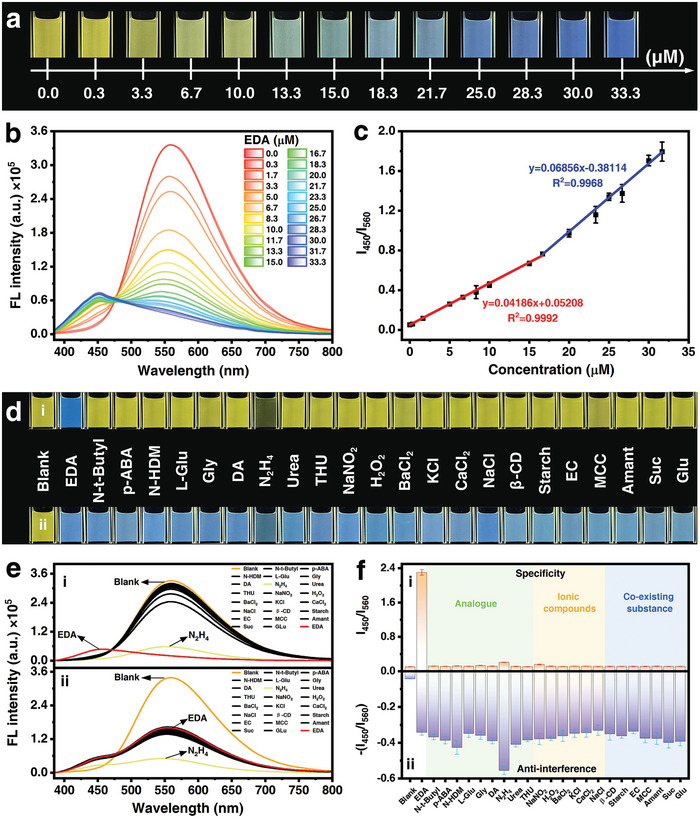
Fluorescence sensing performances of the probe. a) Optical images and b) emission spectra of the BTVB‐DCN probe in response to the increasing EDA (0–33.3 µm), c) the corresponding correlation between the emission intensity ratios at 450 nm to 560 nm (*I*_450_/*I*_560_) and EDA concentrations; Specificity and anti‐interference studies of the BTVB‐DCN probe to EDA (33.3 µm), potential interferents, structural analogs and common ions (0.67 mm): the corresponding d) optical images, e) emission spectra, and f) histogram of the intensity ratio of *I*_450_/*I*_560_ to each analyte. Note: All images and emission spectra were obtained under 365 nm excitation, the error bar represents three experimental replicates. Part analytes were labeled as the following abbreviations, N‐tert‐butylacrylamide (N‐t‐Butyl), *p*‐aminobenzamide (*p*‐ABA), N‐hydroxydiimide (N‐HDM), L‐glutamine (L‐Glu), glycine (Gly), dopamine (DA), hydrazine (N_2_H_4_), urea, thiourea (THU), sodium nitrite (NaNO_2_), hydrogen peroxide (H_2_O_2_), barium chloride (BaCl_2_), potassium chloride (KCl), calcium chloride (CaCl_2_), sodium chloride (NaCl), encapsulant materials *β*‐cyclodextrin (*β*‐CD), starch, ethyl cellulose (EC), microcrystalline cellulose (MCC), amantadine (Amant), sucrose (Suc), glucose (Glu).

To explore whether the BTVB‐DCN probe holds the ability to analyze EDA with high‐quality response, a series of experiments were carried out under the optimized condition of a 10 µm probe dissolved in a mixture solution of DMSO/H_2_O (*v:v* = 2:1) at a pH of 7.0 (Figures S18–S27, Supporting Information), starting with the quantitative detection to EDA solution with the gradient concentrations ranging from 0 to 33.3 µm. It can be seen that the inherent yellow fluorescence of the BTVB‐DCN probe was gradually diminished and transformed to the deepened blue emission along with the increasing EDA concentration under 365 nm illumination (**Figure** **2**a), particularly, the addition of EDA with a concentration of 6.7 µm can be visually distinguished from this ratiometric fluorescence change. Accordingly, when more EDA molecules were added, the probe fluorescence emission at 560 nm exhibited a declining trend, meanwhile, a newly appeared emission at 450 nm gradually strengthened, in which the latter emission indicates the formation of the product (Figure 2b).

The correlation regarding the intensity ratio at 450  to 560 nm (*I*_450_/*I*_560_) versus EDA concentration was further plotted which shows a good linear regression for a two‐segmented calibration curve with fitting coefficients (*R* ^2^) of 0.9992 and 0.9968 (Figure 2c). Based on the classical equation of detection of limit (LOD) defined as LOD = 3*σ*/*k*, the *k* represents the slope of the calibration curve and the σ stands for the standard deviation of the blank solution (*n* = 10), thus, the LOD was calculated as 8.6 nm. This strongly demonstrates that the probe is capable of quantitively detecting EDA with the visualized phenomenon, and the sensitivity satisfies the safety threshold of 8.3 µm for the EDA solution specified by the international authority (e.g., European Union).

In order to explore the specific recognition ability of the probe toward EDA, 22 substances including structural analogs, environmental ions, and other potential co‐existing substances were selected as the interferents to conduct the specificity and the anti‐interferent ability studies. In terms of specificity, 33.3 µm EDA was added to the probe solution while the above‐mentioned interferents with a much higher concentration of 0.67 mm were mixed with probe, respectively. It is obvious that only the existence of EDA could lead to a ratiometric fluorescence change from yellow to deepened blue while other substances cannot induce this specific fluorescence response (Figure 2d(i)). It should be noted that the hydrazine resulted in a quenched fluorescence which is distinctly different from EDA and other substances, suggesting a complete recognition mechanism led to discriminability. The corresponding emission spectra verify the above optical changes as the new emission at 450 nm was observed with the existence of EDA, the intensity at 560 nm was quenched with the addition of N_2_H_4_, while the emission at 560 nm of the probe almost remained unchanged in the cases of other substances (Figure 2e(i)). The histogram of the intensity ratio of *I*_450_/*I*_560_ to each analyte also shows the most outstanding response from the case of EDA, hence, the excellent specificity of the probe to EDA was confirmed (Figure 2f(i)). For the anti‐interferent ability, EDA and the interferents were added to the probe solution together with a volume ratio of 1:1, it can be found that the yellow fluorescence of the probe transformed into blue in all cases due to the existence of EDA, and the emission decreased at 560 nm with the similar intensity ratios of *I*_450_/*I*_560_ (Figure 2d–f(ii). It is clear that the emission decreased more significantly in the mixture of EDA and hydrazine, which can be ascribed to the extra quenching from the existence of hydrazine. Hence, compared with the previously reported optical sensing methods for EDA (Table S3, Supporting Information), the proposed probe holds merits of detecting trace EDA in specific recognition and response time (<1 s, Figure S28, Supporting Information), and is even more outstanding in discriminating EDA and hydrazine simultaneously with two distinctly different optical signals, which can be ascribed to the unique probe design of the two C═C sites with finely different reactivities.

### 2.3 Sensing Performances of the Probe‐Embedded Substrate to EDA Vapor

**Figure 3:**
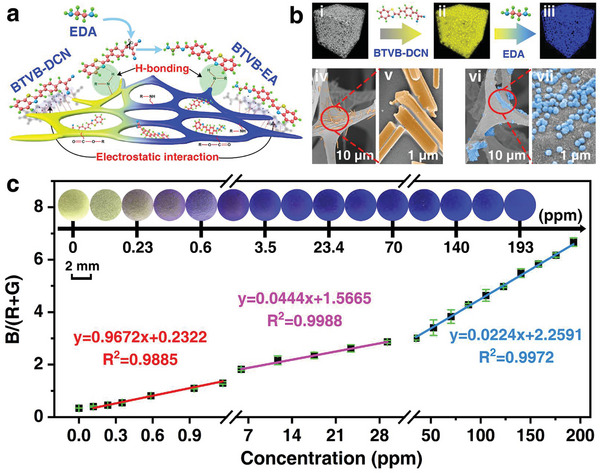
Sensing performances of the probe functionalized substrate. a) Schematic illustration of the multiple interactions between the probe and the polyurethane (PU) substrate; b): i–iii) Schematic illustration of the probe‐embedded PU substrate before and after detecting EDA, iv–vii) the corresponding SEM images of the embedded PU substrate iv,v) before and vi,vii) after interacting with EDA; c) Fluorescence responses of the functionalized substrate toward EDA vapor with a concentration varying from 0 to 193 ppm upon the evaluation of the B/(R+G) value of the fluorescent image as a function of EDA concentration; Note: All images and emission spectra were obtained under 365 nm excitation, the error bar represents three experimental replicates.

Considering EDA easily volatilizes under normal temperature and pressure, the probe was loaded on a solid substrate with a large contact area for better catering to the sensing demand for EDA vapor (**Figure** **3**a). A polyurethane (PU) material was selected as substrate upon the following considerations: i) its hierarchical porous skeleton benefits for adsorbing vapor molecules and inhibiting the diffusion, which could endow a more sensitive response than that in a solution state due to free‐diffusion; ii) it could anchor the probe molecules via non‐covalent interactions, e.g., hydrogen bonding, electrostatic interaction, etc., which could be illustrated by the independent gradient modeling (IGM) analysis and energy dispersive spectroscopy (EDS) analysis (Figures S29 and S30, Supporting Information), further providing more sensing sites. After loading the probe, the PU substrate was shown in yellow fluorescence and its SEM image displayed the rod‐like morphology, while the functionalized substrate was transformed in blue fluorescence in the presence of EDA vapor and the SEM image accordingly showed grainy substances, indicating the successful loading and sensing (Figure 3b). The functionalization and the sensing processes were also confirmed with the presence and the disappearance of the ATR‐FTIR characteristic peaks at 690 and 2220 cm^−1^ of the probe after loading and interacting with EDA vapor (Figure S31, Supporting Information). After interacting with EDA, the ─CH═CH, ─C≡N relevant peaks at 690 and 2220 cm^−1^ were weakened significantly, indicating the C═C in vinyl dicyanide of the probe has reacted with the EDA.

Furthermore, the sensing capability of the functionalized PU substrate was investigated by first placing it in EDA vapor with a concentration ranging from 0 to 193 ppm. It is clear that the optical images show a gradually changed fluorescence from yellow to deepened blue with a naked‐eye LOD of 0.23 ppm (Figure 3c). To better understand the correlation between the concentration gradient of EDA vapor and the optical responses, the RGB values of each image were extracted, and the B/(R+G) value with the best correlation was selected for the fluorescence image processing (closer to 1 than other values, Figure S32, Supporting Information). The B/(R+G) values were plotted as a function of the EDA vapor concentrations, and it shows that the B/(R+G) value was gradually enhanced along with the increasing EDA vapor concentration, and the good linearity for a three‐segmented calibration curve was found with the fitting coefficients of 0.9885, 0.9988 and 0.9972 (Figure 3c; Figure S33, Supporting Information). Accordingly, the LOD was calculated as 1.61 ppb with the *k* = 0.9672 and *σ* = 0.00052, which is definitely superior to that in the reported works and much lower than the occupational exposure limitation of 10 ppm stipulated by WHO, illustrating a desirable sensitivity for meeting a practical need.

To explore the specific recognition ability of the sensing substrate, it was placed in 28 common gases with a concentration of 10^3^ times higher than EDA, and its fluorescence change was recorded under 365 nm excitation (Figure S34, Supporting Information). Similar to the cases in specific studies of detecting solutions, only the existence of EDA could make the yellow fluorescence of the probe transform into blue, the addition of N_2_H_4_ quenched the yellow fluorescence, while all other interferents cannot induce the fluorescence change (Figure S35, Supporting Information). By further histogram analysis, EDA exhibits the highest B/(R+G) value which can be significantly differentiated from other interferents and causes a deepened blue fluorescence in all the mixture gases of interferents and EDA (Figure S36, Supporting Information). Therefore, it indicates that the probe‐embedded sensing substrate still holds the ability to specifically recognize the EDA vapor and discriminate EDA and N_2_H_4_ vapors with completely different responses.

### 2.4 CNN Algorithm Aided Precise Recognition for EDA at Ultra‐Low Concentration

**Figure 4:**
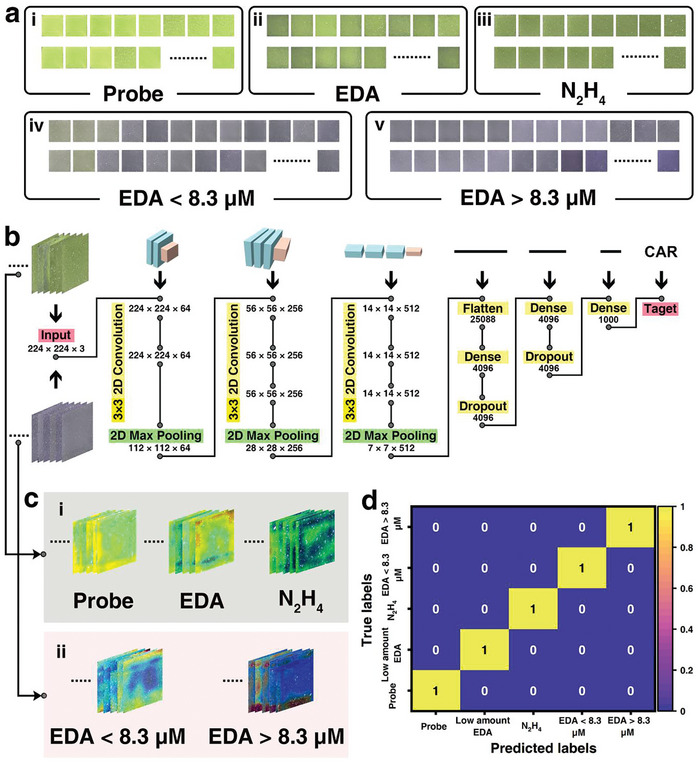
CNN algorithm facilitated the classification of the sensing images. a) Optical sensing images for the probe i) before and after interacting with ii) EDA vapor, iii) hydrazine vapor, and iv) EDA vapor with a concentration lower or v) higher than the threshold of 8.3 µm; b) Schematic illustration of the analysis procedure of the typical CNN algorithm VGG‐16; c) Heat maps of the image classification in the training model, i) the probe, EDA vapor, and hydrazine vapor, and ii) EDA vapor with a concentration higher or lower than the threshold of 8.3 µm; d) Confusion matrix for the classification outcome of the training model.

Moreover, it should be noted that when the EDA vapor was at an extremely low level (e.g., 0.12–1.8 ppm), the sensing images of EDA were fairly approached to the images of the probe itself, and the sensing image of hydrazine at a concentration of 100 ppm, thus, it is difficult to judge whether it is a risk and/or what type of risks is, based on the naked‐eye observation (**Figure** **4**a(i–iii)). One typical CNN algorithm–VGG‐16 with an architecture of 16 layers was applied to process all the above images through a series of convolution and pooling, followed by classifying and outputting the heat map with the extracted crucial image characteristics (Figure 4b). It is obvious the processed heat maps exhibited much more distinct differences between the individual groups than their original sensing images (Figure 4c). Afterward, the clear three groups were received which were treated as the prediction model (Figure 4d), while the unknown sample images were input and auto‐judged by the model, the confusion matrixes were generated according to the classification results for the unknown samples. It was found that all the unknown samples were classified with an accuracy of 100% (Figure S37, Supporting Information), strongly suggesting the combination of this algorithm model was capable of recognizing the EDA at the ultra‐trace level, surpassing the discrimination upon natural vision.

Similarly, to achieve more timely and precise warning, the sensing images for the EDA solution with concentrations around the threshold of 8.3 µm (1.7–8.3 µm EDA solution used in Figure 4a(iv), 8.3–35.1 µm EDA solution used in Figure 4a(v)) were analyzed upon the above VGG16 algorithm model, there also received the precise judgment outcomes (Figure S37, Supporting Information). The results demonstrated that the incorporation of the elaborately designed sensing mechanism and the deep learning algorithm model powerfully lifts the discrimination ability of the proposed sensing strategy for either distinguishing trace EDA from others or judging the level of an unknown EDA concentration relative to the threshold.

### 2.5 Practical Applications Based on a Portable Sensing Chip

**Figure 5:**
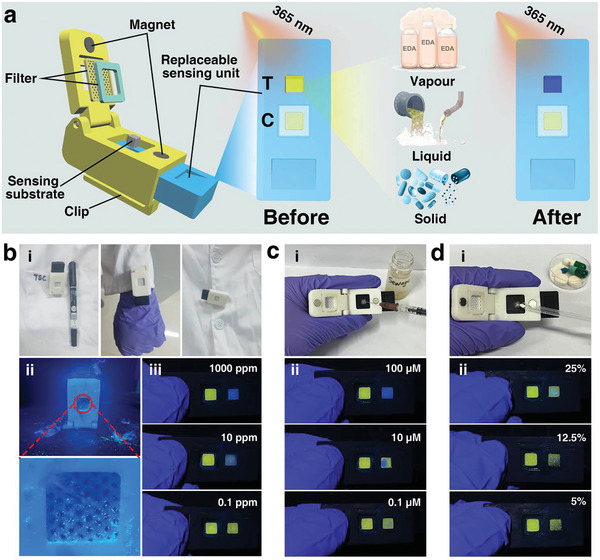
Practical applications of the portable sensing chip in real scenarios. a) Schematic illustration of the portable sensing chip and potential applications for gaseous, liquidus and solidus analytes; b) Potential application for analyzing vaporous EDA by clipping the sensing chip at i) the pocket, the cuffs, and the edge of the clothes, ii) the sensing chip applied in a complicated atmosphere environment, iii) containing different concentrations of EDA vapor; c) Application for analyzing the simulated sewage containing different concentrations of EDA; d) Application for analyzing the simulated medicine containing different contents (*w*/*w*) of EDA.

To further widen the practical applicability, a portable sensing chip was developed by applying a 3D printer to fabricate the shell and the replaceable sensing unit, in which there was a groove to load the probe‐embedded sensing substrate (**Figure** **5**a). The portable sensing chip was expected to be applied for trace EDA detection in multiple practical scenarios, such as the storage garage, industrial sewage, and the assembly line of medication (Figure S38, Supporting Information). Considering the portability and the resistance to environmental co‐existing substances, the skeleton was equipped with a clip for easy carrying and a cover of polytetrafluoroethylene filter (EPTFE, average pore size ≈2.5 µm) for minimizing the influence from the environmental fluorescent substances (e.g., fiber, dust). In reality, the sensing chip can be clamped onto the clothing (e.g., pocket, cuff) of the operator (Figure 5b(i)). To verify the feasibility of the sensing chip, it was placed in a complicated container in which EDA vapor was mingled with some fluorescent interferents, such as sundries and phosphor powders. Ascribing to the separation of the sensing unit from the fluorescent interferents by the filter (Figure 5b(ii)), this chip shows characteristic blue fluorescence in the “Test (T)” region with different concentrations of EDA vapor from 0.1 to 1000 ppm. It can be seen that the sensing chip holds the ability to indicate the presence of EDA at the threshold concentration (10 ppm) and even at a lower concentration (0.1 ppm), verifying the effectiveness of the sensing chip (Figure 5b(iii)).

Furthermore, to expand the applicability, the portable sensing chip was applied to analyze EDA in the form of liquid by simulating the sewage containing EDA (Figure 5c(i)). It could analyze the EDA with the concentration around the threshold (8.3 µm) and was not influenced by the co‐existing soluble and insoluble impurities (e.g., anions, cations, urea, soil) (Figure 5c(ii)). Moreover, as EDA is a primary ingredient for producing some medications and its concentration needs to be monitored to reach a certain content (e.g., 11.25% mass content of EDA required in aminophylline), the analysis of EDA blended with the common pharmaceutical excipients as the medication sample was conducted to simulate the potential application at the assembly line (Figure 5d(i)). It is clearly shown that the sensing chip could visually monitor the presence of EDA in the simulated medicine with the varied content from 5% to 25%, showcasing the ability to analyze the solidus sample and apply it for quality control of medicine manufacturing (Figure 5d(ii)).

## 3 Conclusion

In summary, by making the utmost of the subtle difference in nucleophilicity, a distinctive sensing probe was proposed with two recognition sites to achieve the ultra‐sensitive ratiometric fluorescence response to EDA with discriminable quenching response to hydrazine. It is found that the dual‐nucleophilic attack endowed sensing is highly efficient, for which the probe reacting with EDA at an electron‐deficient region with an ESP of 20.18 kcal mol^−1^ and the hydrazine attacking the other more electron‐deficient site with a higher ESP of 35.42 kcal mol^−1^. Thus, a high sensitivity of ppb/nm level, an undisturbed recognition ability of other interferences, and an instant and visualized signal toward EDA were achieved. By feat of the convolutional algorithm‐based image processing strategy, the fluorescent sensing images which are difficult to discriminate with the unaided eye, were precisely classified. Furthermore, a portable sensing platform was demonstrated with a sensing ability for detecting the EDA in liquidous, gaseous, and solidus samples with desirable sensitivity. We expect this innovative sensing strategy would help to open up the possibility of achieving the multi‐target analysis for early warning and disposal in many fields, e.g., medicine, public security, and environmental monitoring.

## 4 Experimental Section

### Materials

Unless otherwise specified, the reagents and materials were obtained from commercial sources and used as received. Polyurethane (PU) substrate with a size of 3 × 3 × 3 mm was acquired from Shenzhen Jiexin Rubber & Plastic Sponge Products Co., Ltd., while the photosensitive resin for 3D printing was purchased from Beijing Weikeng Rui Bo Technology Co. Ltd. Organic chemicals, including 2‐methylbenzothiazole, terephthalaldehyde, malononitrile, acetic anhydride, acetic acid, methanol (MeOH), acetonitrile (ACN), trichloromethane (TCM), dichloromethane (DCM), ethyl acetate (EA), dimethyl ketone (DMK), *N*,*N*‐dimethylformamide (DMF), tetrahydrofuran (THF), dimethyl sulfoxide (DMSO), petroleum ether (PE), anhydrous ethanol (EtOH), and other chemicals, e.g., hydrochloric acid (HCl), and sodium hydroxide (NaOH), were purchased from Aladdin Reagent Co., Ltd. All the reagents were analytical and chromatographic grade.

### Characterizations

By using tetramethyl silane as the internal standard and DMSO‐*d*_6_ as deuterium solvent, the ^1^H NMR and ^13^C nuclear magnetic resonance (NMR) spectra were collected through a high‐resolution 400 MHz NMR spectrometer (Burker, Germany). The mass spectra were measured with a Q Exactive‐type four‐stage rod Orbitrap high‐resolution mass spectrometer (HRMS, UHPLC‐Q‐Orbitrap‐HRMS, Thermo Fisher Scientific, USA) and an Agilent Technologies 6530 TOF LC/MS (Agilent, Japan). The fluorescence spectra were collected on an Edinburgh FLS1000 fluorescence spectrophotometer (Edinburgh Instruments, UK). The colorimetric and fluorescent images were obtained by the iPhone 13 mini (Apple Inc., USA) or captured by an industrial camera (Vision Datum Mars 5000S‐20gc). Field‐emission scanning electron microscopy (FE‐SEM, JEOL JSM‐7610 F Plus, Japan) with a voltage of 4.0–6.0 kV was used for the morphology characterization of sensing substrate. Attenuated total reflection Flourier‐transformed infrared (ATR‐FTIR) spectra and Fourier‐transform infrared (FT‐IR) spectra were obtained by a PerkinElmer Frontier with a universal ATR sampling accessory from PerkinElmer (PerkinElmer, Japan). The RGB values were extracted using the software of Adobe Photoshop 2022.

## Conflict of Interest

The authors declare no conflict of interest.

## Supporting information

Supporting Information

## Data Availability

The data that support the findings of this study are available from the corresponding author upon reasonable request.
