# Supplementary material for: Electrophilicity Modulation for Sub‐ppm Visualization and Discrimination of EDA
Source: Adv Sci (Weinh). 2024 Mar 6;11(18):2400361. doi: 10.1002/advs.202400361 (PMC11095169; doi:10.1002/advs.202400361)
Supplement: Supplementary file 1 — Supporting Information [file ADVS-11-2400361-s001.pdf]

ADVANCED SCIENCE
Open Access

Supporting Information

for *Adv. Sci.*, DOI 10.1002/advs.202400361

Electrophilicity Modulation for Sub-ppm Visualization and Discrimination of EDA

Hao Zhao, Yuan Liu, Gaosheng Li, Da Lei, Yuwan Du, Yudong Li, Hui Tang* and Xincun Dou**

Supporting Information

Electrophilicity Modulation for Sub-ppm Visualization and Discrimination of EDA

Hao Zhao, Yuan Liu, Gaosheng Li, Da Lei, Yuwan Du, Yudong Li, Hui Tang*, Xincun Dou**

Supplementary experimental details

Synthesis of the probe

The synthetic route for 2-(4-(2-(benzo[d]thiazol-2-yl)vinyl)benzylidene)malononitrile (BTVB-DCN) is shown in scheme S1: 2.26 mL 2-methylbenzothiazole and 2.6972 g terephthalaldehyde were added into 20 mL acetic anhydride with a molar ratio of 1:1, followed by the dropwise addition of 3 mL acetic acid and the reaction at 120 °C in a nitrogen atmosphere for 12 hours. Then, a colour change from colourless to light yellow of the reaction solution was observed.

Afterwards, 30 mL 37.5% hydrochloric acid were added slowly into the reaction solution, accompanying with a colour change of the solution from light yellow to reddish-brown and the presence of black precipitate. After the filtration, 30% NaOH solution (~ 60 mL) was added to the filtrate and the light yellow precipitate was received, which was washed with plenty of deionized water to obtain the crude product of yellow needle-shaped crystals. The crude product was further purified by the column chromatography using PE:EA with a volume ratio of 10:1 ratio and the recrystallisation, the desired intermediate product of 4-(2-(benzo[d]thiazol-2-yl)vinyl)benzaldehyde (BTVB-CHO) was obtained with a yield of 78.46 % and the morphology of needle-like crystal.[1, 2, 3] ^{1}H NMR (400 MHz, DMSO-d_6) δ (ppm): 10.03 (s, 1H), 8.12 (d, J = 8.0 Hz, 1H), 8.04 – 7.93 (m, 5H), 7.86 – 7.73 (m, 2H), 7.58 – 7.44 (m, 2H).^{13}C NMR (101 MHz, DMSO-d_6) δ (ppm): 192.99, 166.31, 153.92, 141.43, 136.71, 136.35, 134.80, 130.44, 128.71, 127.15, 126.24, 125.31, 123.28, 122.7. APCI-MS m/z: $[M+H]^+$ calculated for 266.0634; Found 266.0623.

200 mg intermediate product and 112 mg malononitrile were added to 50 mL EtOH, the mixture was stirred at 80 °C for 12 hours. Subsequently, a solidus crude product in orange was obtained, which was purified by column chromatography by using DCM as the eluent. The desired product of 2-(4-(2-(benzo[d]thiazol-2-yl)vinyl)benzylidene)malononitrile (BTVB-DCN) with a yield of 76.58 % was obtained. ^{1}H NMR (400 MHz, DMSO-d_6) δ (ppm): 10.03 (s, 1H), 8.12 (dd, J = 8.0, 1.3 Hz, 1H), 8.05 – 7.90 (m, 6H), 7.87 – 7.64 (m, 2H), 7.59 – 7.51 (m, 1H), 7.47 (td, J = 7.6, 1.3 Hz, 1H).^{13}C NMR (101 MHz, DMSO-d_6) δ (ppm): 166.20, 160.81,

153.93, 141.38, 136.00, 134.85, 132.18, 131.59, 128.93, 127.22, 126.35, 125.83, 123.33, 122.82, 113.87, 81.71. APCI-MS m/z: $[M+H]^+$ calculated for 314.0746; Found 314.0728.

Preparation and characterization of single crystal of the probe

The X-ray quality single crystal of BTVB-DCN was grown by slow evaporation of its saturated DCM solution at room temperature. The probe BTVB-DCN crystals were mounted in random directions on the glass fibre. Single crystal of $C_{19}H_{11}N_3S$ was shown in Figure S9 and the detailed information was summarized in Table S1. A suitable crystal was selected and on a Bruker D8 VENTURE TXS PHOTON 100 diffractometer. The crystal was kept at 293.10 K during data collection. Using Olex2,[4] the structure was solved with the SHELXT[5] structure solution program using Intrinsic Phasing and refined with the SHELXL[6] refinement package using Least Squares minimization.

Optimization of solvent type of the probe

The BTVB-DCN probe was dissolved in various solvents, MeOH, ACN, TCM, DCM, EA, DMK, DMF, THF and DMSO, respectively, to form a solution with a concentration of 1 mM, and fluorescence images and spectra were recorded. 200 µL 10 mM EDA solution was mixed with 400 µL the above-mentioned probe solution, respectively. After completed reaction, the fluorescence images and spectra of the reaction solution were recorded. The fluorescence spectra were recorded with a setting of λ_{ex}= 365 nm, slits = 1.5 nm, λ_{em} = 450 nm, slits = 1.5 nm.

Optimization of the volume ratio of the mixed DMSO/H_2O for probe

The BTVB-DCN probe was dissolved in different ratios (1:0, 5:1, 4:1, 3:1, 2:1, 1:1, 1:2, 1:3, 1:4, 1:5, 0:1) of the mixed DMSO/H_2O, then, the fluorescence images and spectra were recorded with a setting of λ_{ex}= 365 nm, slits = 1.5 nm, λ_{em} = 560 nm, slits = 1.5 nm.

Optimization of the concentration of the probe

The BTVB-DCN probe was dissolved in DMSO with a series of concentrations of 1, 5, 10, 20, 30 and 40 µM, followed by the mixture of 400 µL probe and 200 µL 1 mM EDA solution. After the reaction, the fluorescence images and spectra were recorded with a setting of λ_{ex}= 365 nm, slits = 1.5 nm, λ_{em} = 450 nm, slits = 1.5 nm.

Optimization of the pH of the probe

To gain a buffer with the pH of 0.4, 0.338 mL 36.5% HCl was added to 10mL deionized water and diluted to a series of solutions with the pH values of 1.4, 2.4, 3.4, and 4.4. The

deionized water was used as the buffer with the pH of 7.0. To create a buffer with pH 13.6, 159.6 mg NaOH was dissolved in deionized water and diluted to a series of solutions with the pH values of 12.6, 11.6, 10.6 and 9.6. Then, 390 µL 10 µM probe solution was mixed with 10 µL buffer solution, respectively. The fluorescence images and spectra were recorded with a setting of λ_{ex}= 365 nm, slits = 1.5 nm, λ_{em} = 560 nm, slits = 1.5 nm.

Optimization of the pH of the detection environment

To obtain a buffer with the pH of 0.2, 0.508 mL 36.5% HCl was added to 10 mL deionised water and diluted to a series of solutions with the pH values of 1.2, 2.2, 3.2, and 4.2. The deionized water was used as the buffer with the pH of 7.0. To obtain a buffer with the pH of 13.8, 239.6 mg NaOH in deionized water and diluted to a series of solutions with the pH values of 12.8, 11.8, 10.8 and 9.8. Then, 390 µL 10 µM probe solution was mixed with 10 µL buffer solution and 200 µL 100 µM EDA solution, respectively. The fluorescence images and spectra were recorded with a setting of λ_{ex}= 365 nm, slits = 1.5 nm, λ_{em} = 450 nm, slits = 1.5 nm.

Stability study

10 µM probe solution was stored at temperatures of 10, 20, 30, and 40°C for 7 days. Afterwards, the fluorescence images and spectra were recorded on each single day with a setting of λ_{ex}= 365 nm, slits = 1.5 nm, λ_{em} = 560 nm, slits = 1.5 nm.

400 µL 10 µM probe solution was mixed with 200 µL 100 µM EDA, and then, the mixture was stored at temperatures of 10, 20, 30, and 40°C for 7 days. Afterwards, the fluorescence images and spectra were recorded on each single day with a setting of λ_{ex}= 365 nm, slits = 1.5 nm, λ_{em} = 450 nm, slits = 1.5 nm.

The probe solutions with a series of concentrations of 1, 5, 10, 15, 20, 25, 30, 35, 40, 45 and 50 µM were stored at room temperature for 7 days. Afterwards, the fluorescence images and spectra were recorded on each single day with a setting of λ_{ex}= 365 nm, slits = 1.5 nm, λ_{em} = 560 nm, slits = 1.5 nm.

Repeatability study

2 mL 100 µM probe was mixed with 1 mL 1 mM EDA, after the reaction, the fluorescence images and spectra were recorded on each single day with a setting of λ_{ex}= 365 nm, slits = 1.5 nm, λ_{em} = 450 nm, slits = 1.5 nm. The above procedures were repeated for 8 times to evaluate the detection repeatability.

Construction of the probe functionalized PU substrate

The PU substrate with a size of 3×3×3 mm and an average pore size of 2.5 μm was immersed into 10 μM probe solution for a couple of minutes, then, the probe functionalized PU substrate was obtained for future usage.

Sensitivity study

For the EDA solution test: 10 mM EDA solution was prepared as the stock solution, then, it was diluted as a series of 0.3, 1.7, 3.3, 5.0, 6.7, 8.3, 10.0, 11.7, 13.3, 15.0, 16.7, 18.3, 20.0, 23.3, 25.0, 26.7, 28.3, 30.0, 31.7, 33.3 μM. 200 μL the above diluted EDA solution was mixed with 400 μL 10 μM probe solution for the detection reaction. The fluorescence images and spectra were recorded with a setting of λ_{ex}= 365 nm, slits = 1.5 nm, λ_{em} = 450 nm, slits = 1.5 nm.

For the EDA vapour test: 0.1 mL 99.5% EDA was placed in 500 mL sealed flask which was subsequently heated to 120 °C, then, the saturated EDA vapour was received. A certain amount of saturated vapour was injected into 250 mL flask by a syringe, the stock vapour was received with a concentration of 878 ppm. A series of diluted EDA vapour samples (0.12, 0.23, 0.35, 0.59, 0.94, 1.17, 1.75, 3.51, 5.85, 11.7, 17.5, 23.4, 29.2, 35.1, 52.6, 70.2, 87.8, 105.4, 122.9, 140.4, 158.0, 175.6, 193.1 ppm) were obtained by injecting different volumes of the stock vapour into different volumes of air. The probe functionalized PU substrate was placed in a sealed flask which was filled with the above-diluted EDA vapours. After 90 s, the fluorescence image change of the functionalized PU substrate was recorded under a 365 nm UV lamp by an industrial camera.

Specificity study

For the EDA solution test: 32 types of potential interferents including structural analogue, ionic compound and co-existing substance (*e.g.*, pharmaceutical excipient) were selected for evaluating the specific recognition ability of the probe towards EDA, *e.g.*, N-tert-butylacrylamide (N-t-Butyl), *p*-aminobenzamide (*p*-ABA), N-hydroxydiimide (N-HDM), L-glutamine (L-Glu), glycine (Gly), dopamine (DA), hydrazine (N_2H_4), urea, thiourea (THU), diethylamine (DEA), phenethylamine (PEA), urotropine (UTP), phenylhydrazine (PHA), aniline, *o*-phenylenediamine (*o*-PDA), diphenylamine (DPA), acrylamide (AM), N,N-dimethylformamide (DMF), *p*-nitrobenzohydrazide (*p*-NHD), sodium nitrite ($NaNO_2$), hydrogen peroxide (H_2O_2), barium chloride ($BaCl_2$), potassium chloride (KCl), calcium chloride ($CaCl_2$), sodium chloride (NaCl) encapsulant materials *β*-cyclodextrin (*β*-CD), starch, ethyl cellulose (EC), microcrystalline cellulose (MCC), amantadine (Amant), sucrose (Suc), glucose (Glu). All the interferents were prepared in deionized water with a concentration of 2 mM.

200 μL 100 μM EDA or 200 μL 2 mM potential interferents were mixed with 400 μL 10 μM probe solution, then, the fluorescence images and spectra were recorded with a setting of λ_{ex}= 365 nm, slits = 1.5 nm, λ_{em} = 450 nm, slits = 1.5 nm.

For the EDA vapour test: 28 types of potential interferents including common organic vapour, volatile structural analogue, and co-existing vapour were selected for evaluating the recognition specificity of the functionalized PU substrate towards EDA vapour, *e.g.*, dichloromethane (DCM), N,N-dimethylformamide (DMF), ethyl acetate (EA), petroleum ether (PE), acetone (AC), ethanol (EtOH), chloroform ($CHCl_3$), benzene (BZ), styrene monomer (SM), formaldehyde (HCHO), hydrofluoric acid (HF), acetic acid (Hac), formic acid (FA), trimethylamine (TTA), aniline, hydrazine (N_2H_4), thiourea (THU), urea, hydrogen peroxide (H_2O_2), kerosene (KS), petrol, essential balm (EB), safflower oil (SO), floral water (FW), essential oil (EO), fragrance (FA), eyedrops (ED), and disinfectant (DF). All the interferent vapours were prepared at a concentration of 2.0×10^6 ppm for the subsequent measurement.

The probe functionalized PU substrate was placed in a sealed flask which was filled with 878 ppm EDA vapour or 2.0×10^6 ppm potential interferent vapours. After 90 s, the fluorescence image change of the functionalized PU substrate was recorded under a 365 nm UV lamp by an industrial camera.

Anti-interference study

For the EDA solution test: 100 μL 100 μM EDA was firstly mixed with 100 μL 2 mM the above prepared potential interferents, then, the mixture was added into 400 μL 10 μM probe solution. The fluorescence images and spectra were recorded with a setting of λ_{ex}= 365 nm, slits = 1.5 nm, λ_{em} = 450 nm, slits = 1.5 nm.

For the EDA vapour test: The probe functionalized PU substrate was placed in a sealed flask which was filled with the mixture vapours containing 878 ppm EDA vapour and 2.0×10^6 ppm potential interferent vapours. After 90 s, the fluorescence image change of the functionalized PU substrate was recorded under a 365 nm UV lamp by an industrial camera.

Construction of the portable sensing chip

A 3D printing system was applied to fabricate the sensing chip and the replaceable sensing unit as designed skeletion. Then, the functionalize PU substrate was placed in the test region and control region. It should be noted that the control region was sealed with a transparent thermoplastic film to avoid the fluoresence change due to the presence of vapour samples.

Acquisition and data processing of the fluorescent images and videos

All fluorescent images and videos with the actual pixel size of 0.005225 mm were taken by an industrial camera with fitted lens under irradiation of 365 nm UV lamp. The fluorescence changes were recorded in video by an EV Recording software, and then, the images were extracted by frames with Format Factory software. The RGB values of each image was extraced by the Adobe Photoshop software.

Image processing upon the CNN algorithms -- VGG-16

A total of 470 images were acquired to create a CNN classification dataset and randomly divided into two parts with a ratio of 7:3 as the training model and testing model. Then, all images were resized with a resolution of 256×256 during the training and testing process. The VGG-16 algorithm architecture consists of input layer, convolutional layer, pooling layer, fully connected layer, and output layer, which was served as the foundation for classifying images. Firstly, the convolutional neural network was applied to extract the key features from the image while the SGD optimization algorithm was applied for tranining process with a learning rate of 0.0001. Then, the momentum was set to 0.9 and the weight decay was set as 2e-5. As the number of epoch increases, the loss of the difference between prediction outcome and actual outcome more approach to 0, thus, the training model is more accurate. In this case, after 30 epoches, the training model was built. Furthermore, the testing images were input the algorithm, upon the training model, the classification of the testing images were generated.

Analyses of the samples in applicable scenarios

Vapourous sample preparation and test: EDA vapours with concentrations of 0.1, 10, and 1000 ppm was prepared as the above-mentioned method, then, 5 mL other possible interfering atmospheres with a concentration of 1×10^6 ppm (formaldehyde, dichloromethane, ethyl acetate, hydrofluoric acid, trimethylamine, aniline, hydrogen peroxide) and potential co-existing fluorescent substances (4-boronic acid pinacol ester-1,8 naphthalene dicarbonate powder, 2-(2-hydroxyphenyl)benzothiazole powder, cotton wool) were added in. The functionalized sensing chip was placed in the above atmosphere samples for 90 s, then, the sensing chip was illminated under 365 nm UV light and the correpsonding fluorescence images at “T” region were recorded by the iPhone 13 mini.

Liquidous sample preparation and test: The simulated sewage samples containing EDA with the concentrations of 0.1, 10, 100 μM and potential co-existing substances (2 mM soluable NaCl, $BaCl_2$, urea, thiourea, glucose, dopamine, and insoluble impurities 50 mg silica gel powder, 100 mg magnet) were prepared. 0.03 mL sewage sample was injected at “T” region, at

which the fluorescent change was recorded by the iPhone 13 mini under illmination under 365 nm UV light.

Solidious sample preparation and test: The simulated medicine was prepared by blending EDA-silica gel powder with the concentrations of 5% (1 mg), 12.5% (2.5 mg), 25% (5 mg) with the common pharmaceutical excipients including 1 mg NaCl, 1 mg starch, 1 mg cellulose, 1 mg glucose and a certain amount of silica gel powder. 2 mg mixed powder was added at "T" region, at which the fluorescent change was recorded by the iPhone 13 mini under illmination under 365 nm UV light.

Theoretical calculations

All the theoretical calculations were carried out with the Gaussian 09 program.[7] Based on density functional theory (DFT-D3(BJ)),[8] PBE0 exchange correlation functional[9] and def2-SVP basis set were used to optimize the geometry and find the transition state configuration, and def2-TZVP was used for single point energy calculation.[10, 11] In order to simulate the absorption, the time dependent density functional theory (TD-DFT)[12] calculations were carried out to obtain the vertical excitation energies for 30 lowest singlet transitions.The D3 version of Grimme's dispersion was included to describe the weak interaction.[13] The solvent effects were based on the polarizable continuum model (PCM) with DMSO:H_2O (2:1) as the solvent environment.[14, 15] All the optimized structures do not have virtual vibration frequency as confirmed by the vibration analysis calculations. The electrostatic potential (ESP)[16] diagrams showcased reaction site parameters, frontier molecular orbitals (FMOs), analogue spectroscopy (AS), independent gradient model (IGM)[17] of theory described hydrogen bonding interactions, and hole-electron analysis.[18] The drawing of various visual iso-surfaces or plane maps were obtained by the approach proposed by Multiwfn software[19] and rendered by VMD visualization program.[20] Various thermodynamic data were conveniently calculated by using Shermo in conjunction with a quantum chemistry program, outputting various thermodynamic quantities under the default temperature of 298.15 K and pressure of 1 atm.[21]

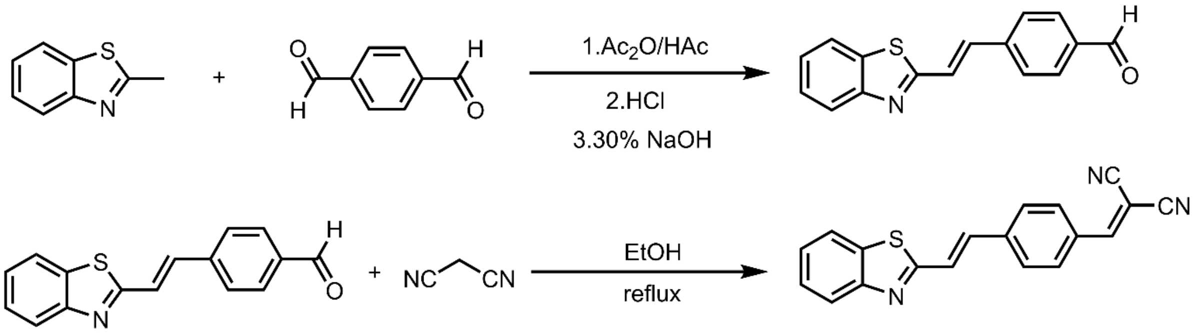

Scheme S1. The synthetic routes of the probe BTVB-DCN.

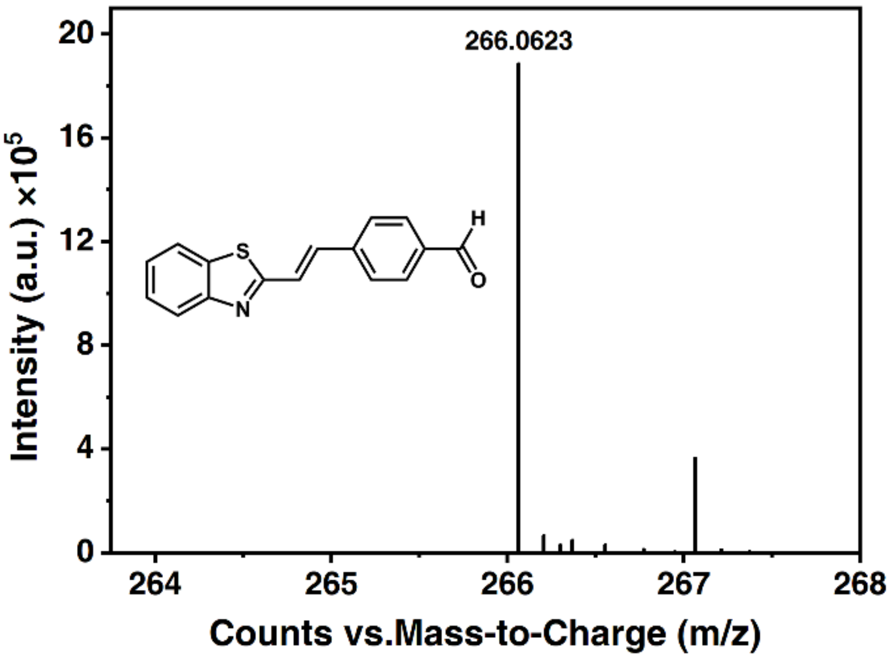

Figure S1. HRMS spectrum of the intermedium compound BTVB-CHO.

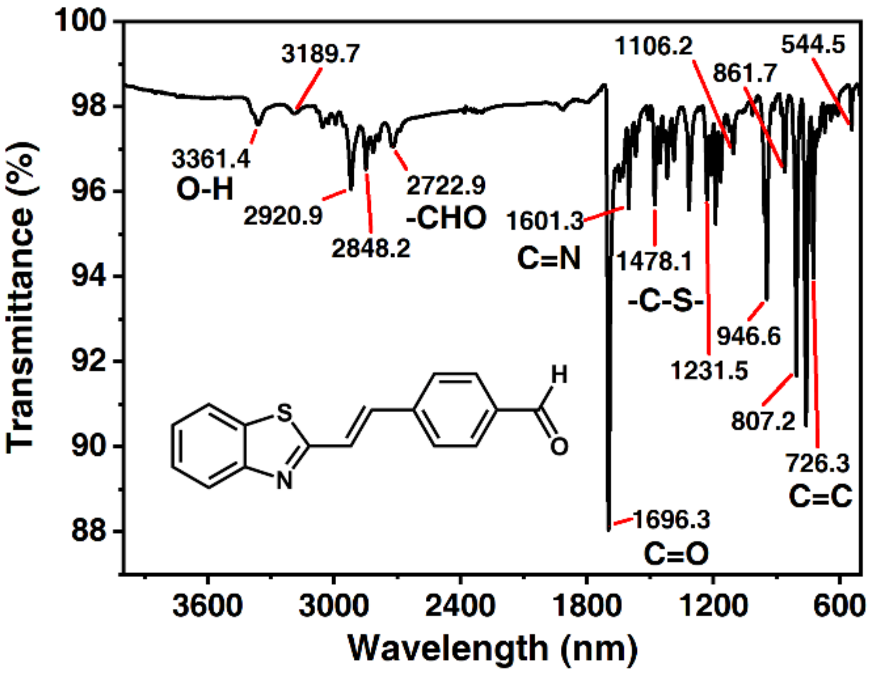

Figure S2. FT-IR spectrum of the intermedium compound BTVB-CHO.

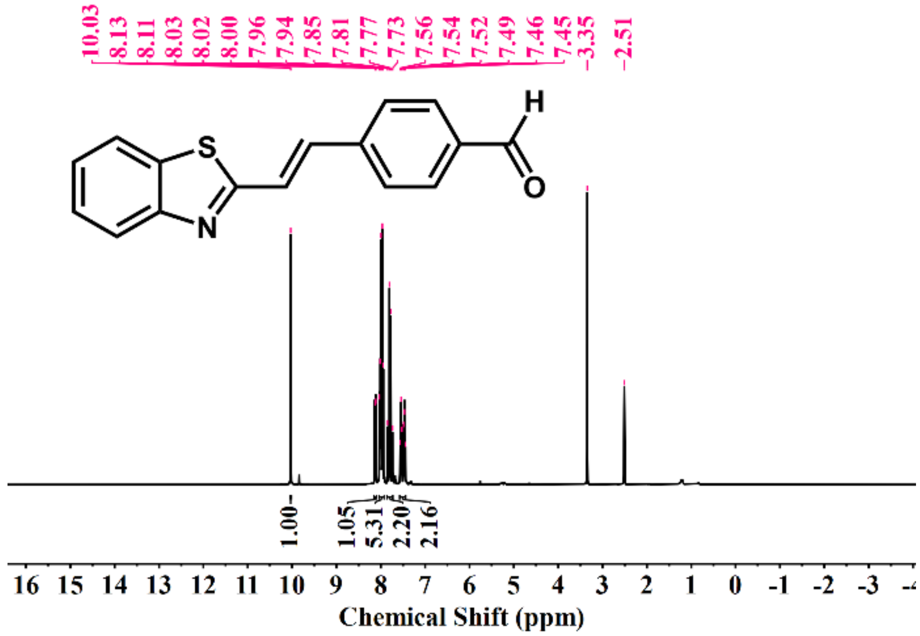

Figure S3. ^{1}H NMR spectrum of the intermedium compound BTVB-CHO.

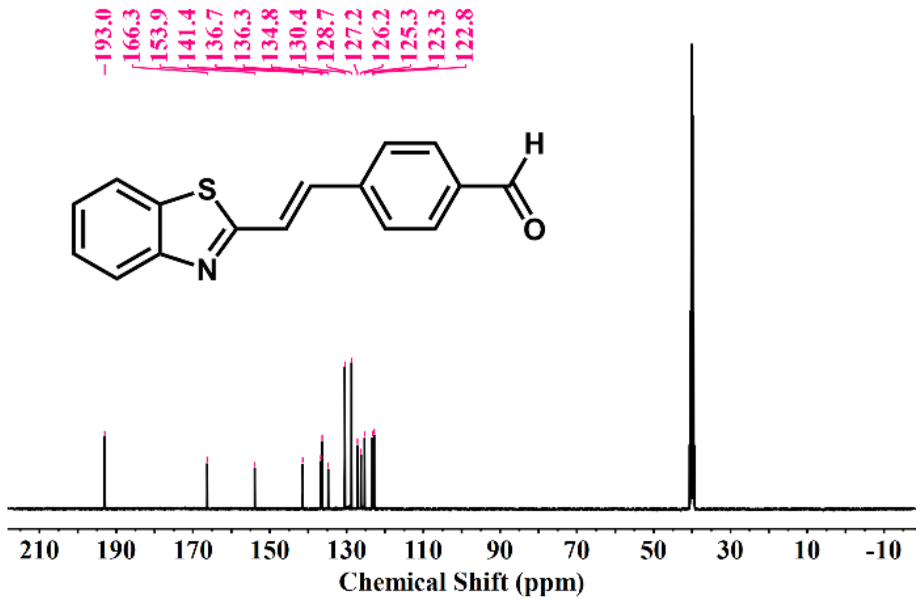

Figure S4. ^{13}C NMR spectrum of the intermedium compound BTVB-CHO.

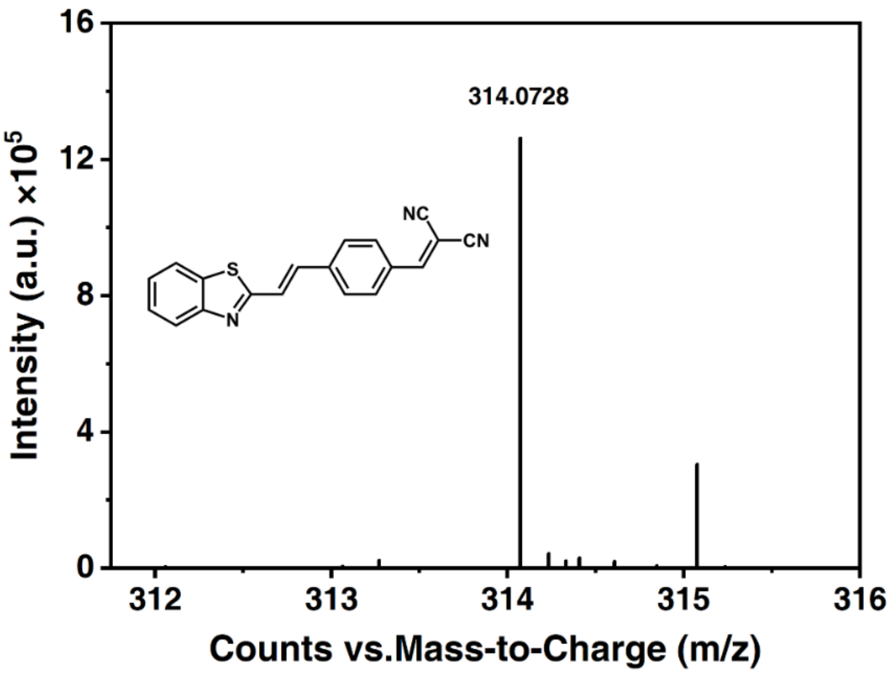

Figure S5. HRMS spectrum of the probe BTVB-DCN.

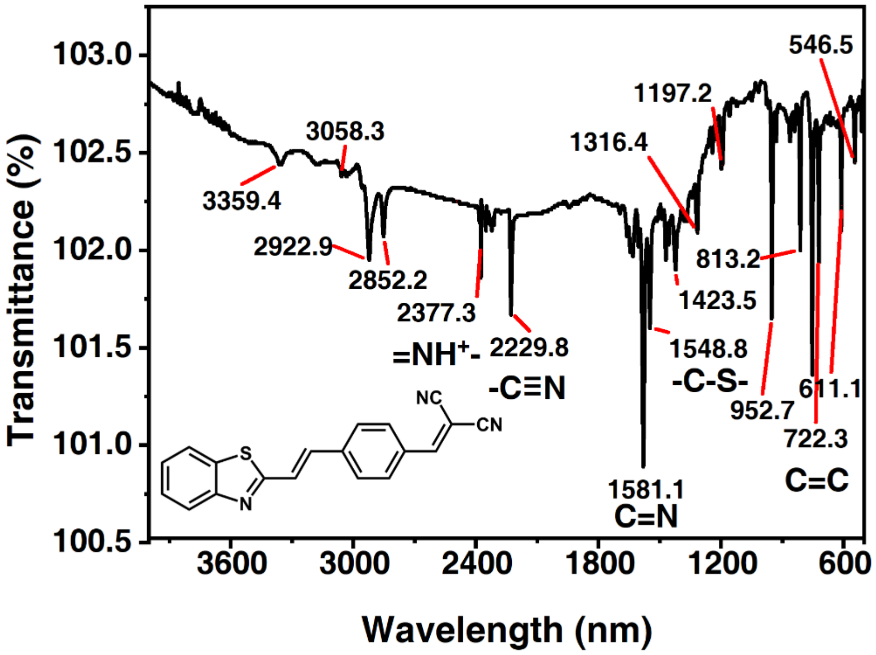

Figure S6. FT-IR spectrum of the probe BTVB-DCN.

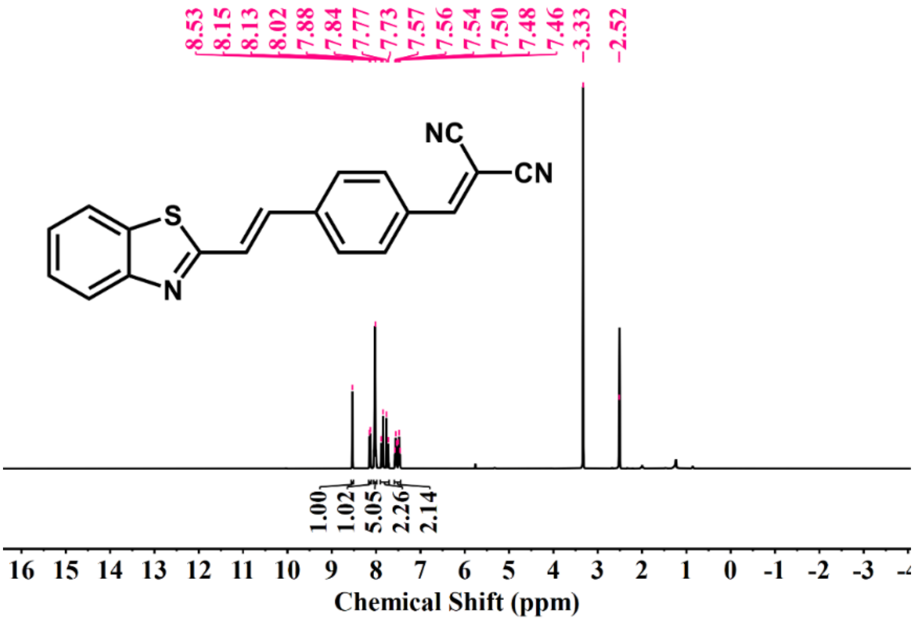

Figure S7. ^{1}H NMR spectrum of the probe BTVB-DCN.

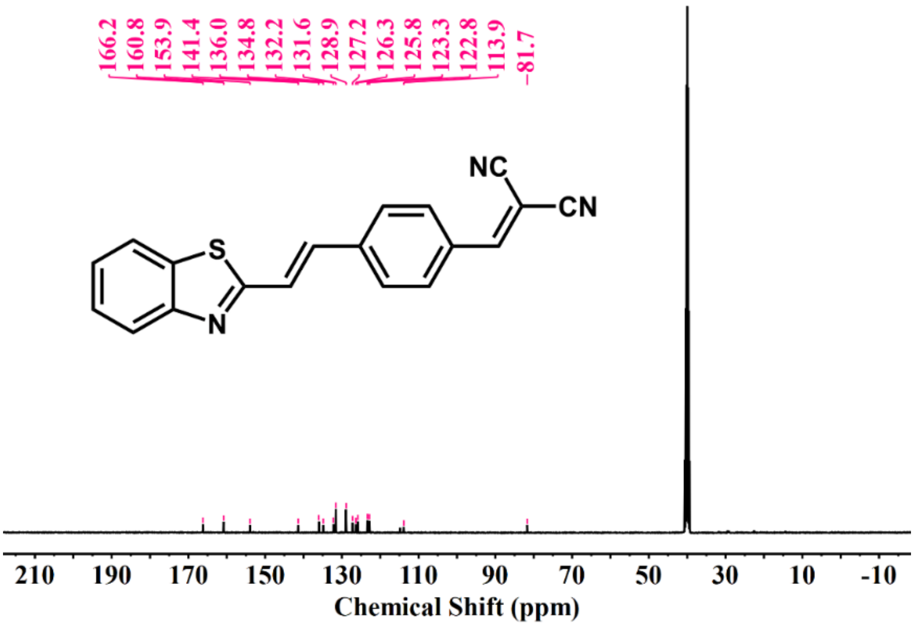

Figure S8. ^{13}C NMR spectrum of the probe BTVB-DCN.

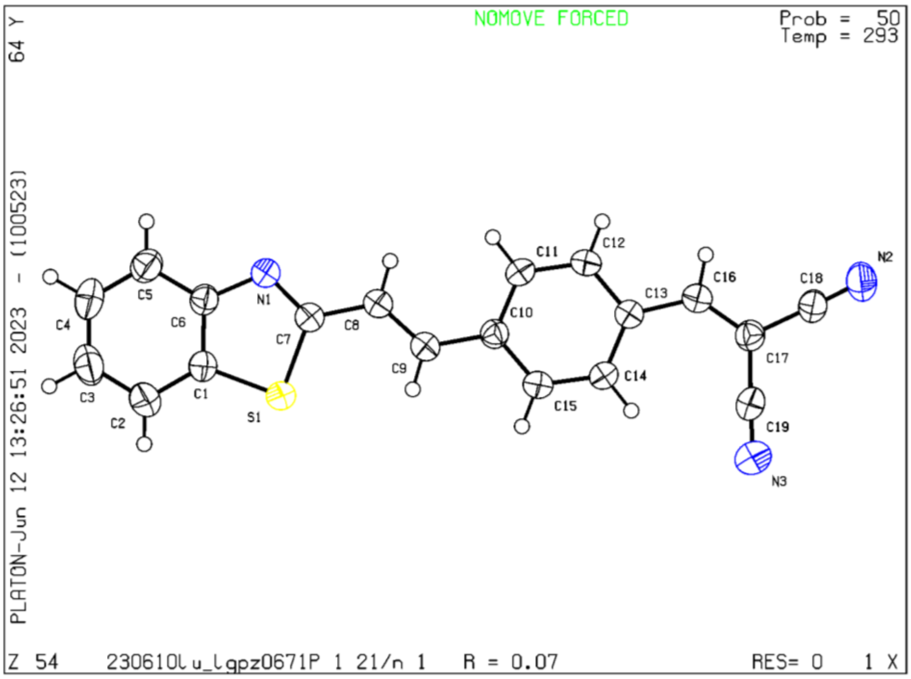

Figure S9. The single crystal structure of the probe BTVB-DCN.

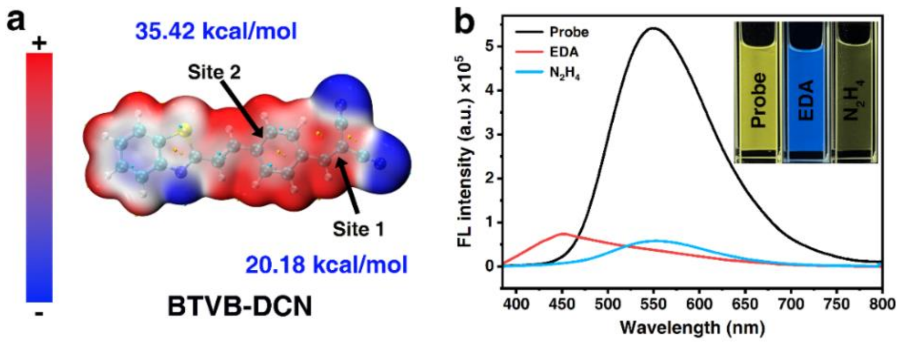

Figure S10. Optical responses of the probe BTVB-DCN towards EDA and hydrazine. (a) ESP analysis of the probe BTVB-DCN; (b) Fluorescence spectra (excited at 365 nm) and the corresponding images (under 365 nm illumination) of the probe before and after detecting EDA and N_2H_4.

Note: The designed BTVB-DCN probe has two relatively positive regions (red) around the carbon-carbon double bonds (C=C) at site 1 and site 2 of the probe, showing the maximum ESP values of 20.18 kcal/mol and 35.42 kcal/mol, respectively. The potential reaction site concentrated at two C=C bonds, and the site 1 at the tail was more electron-deficient, suggesting the site 1 was preferentially attacked by nucleophilic molecule.

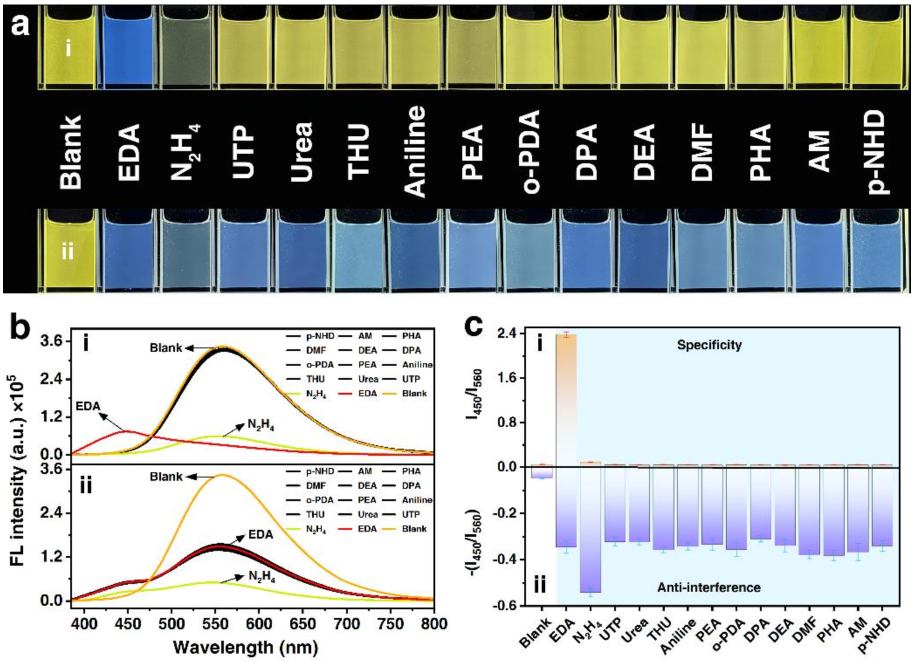

Figure S11. Sensing responses of the BTVB-DCN probe to EDA (33.3 μM), the common hazardous organic amines (0.67 mM). (a) Optical images, (b) emission spectra, and (c) histogram of the intensity ratio of I_{450}/I_{560} to each analyte; i) the analyte was separately added in the probe, while ii) the mixture of EDA and the other organic amine with a molar ratio of 1:20, was added in the probe.

Note: All images and emission spectra were obtained under 365 nm excitation, the error bar represents three experimental replicates. Part analytes were labelled with the abbreviations: hydrazine (N_2H_4), diethylamine (DEA), phenethylamine (PEA), urotropine (UTP), phenylhydrazine (PHA), aniline, *o*-phenylenediamine (*o*-PDA), diphenylamine (DPA), urea, acrylamide (AM), N,N-dimethylformamide (DMF), thiourea (THU), *p*-nitrobenzohydrazide (*p*-NHD).

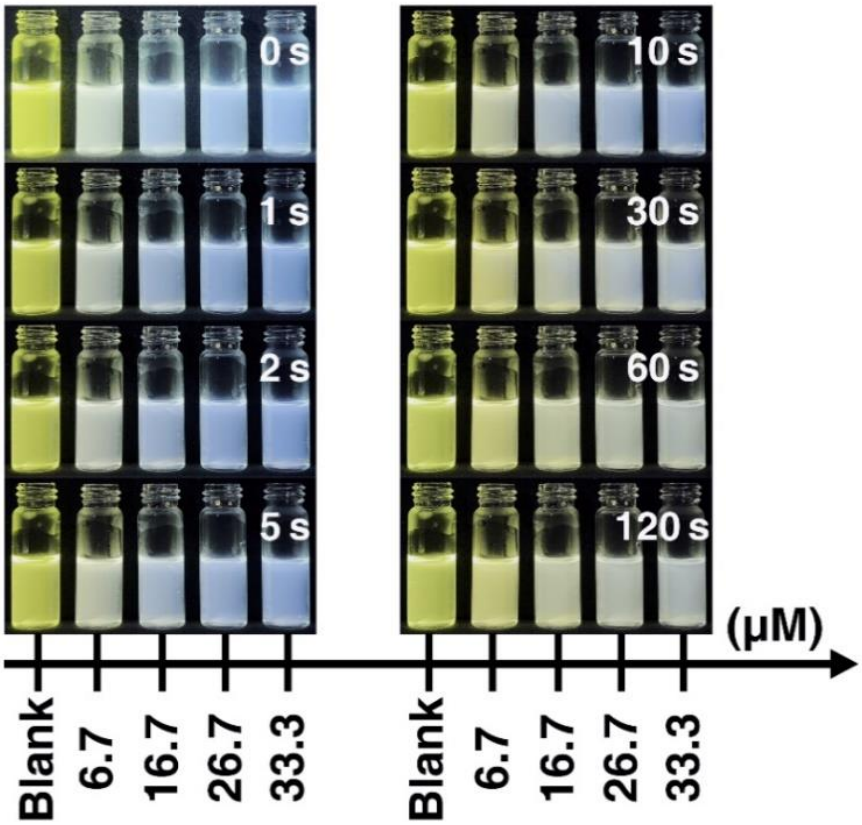

Figure S12. Optical images under 365 nm illumination along with time change for the probe interacting with EDA at different concentrations.

Note: It is clear that under 365 nm UV light, the fluorescence of the reaction product did not change significantly within 5 s after the probe detecting EDA, but the fluorescence was not stable after 10 s. It can be explained as that the imide structure in the amino-nucleophilic attack product is quite unstable as normally recognized,[22, 23] thus, the reaction product cannot be separated effectively.

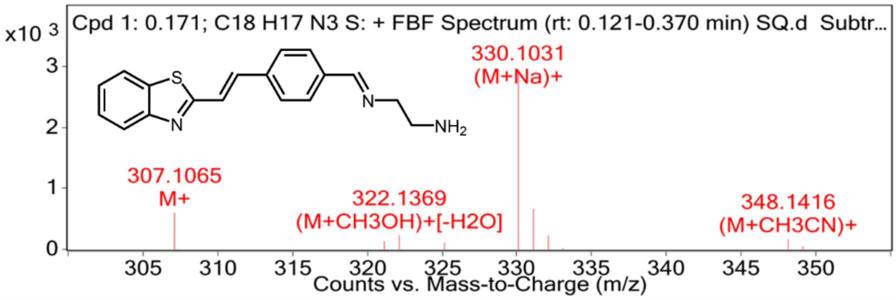

Figure S13. HRMS spectrum of the hypothesized product 2-((-4-(-2-(benzo[d]thiazol-2-yl)vinyl)benzylidene)amino)eth-an-1-amine (BTVB-EA).

Note: The peak of 330.1031 (m/z) (M+Na$^+$) indicates the recognition reaction occurred at site 1, accompanying with the addition of the EDA and the leaving of dicyanovinyl.

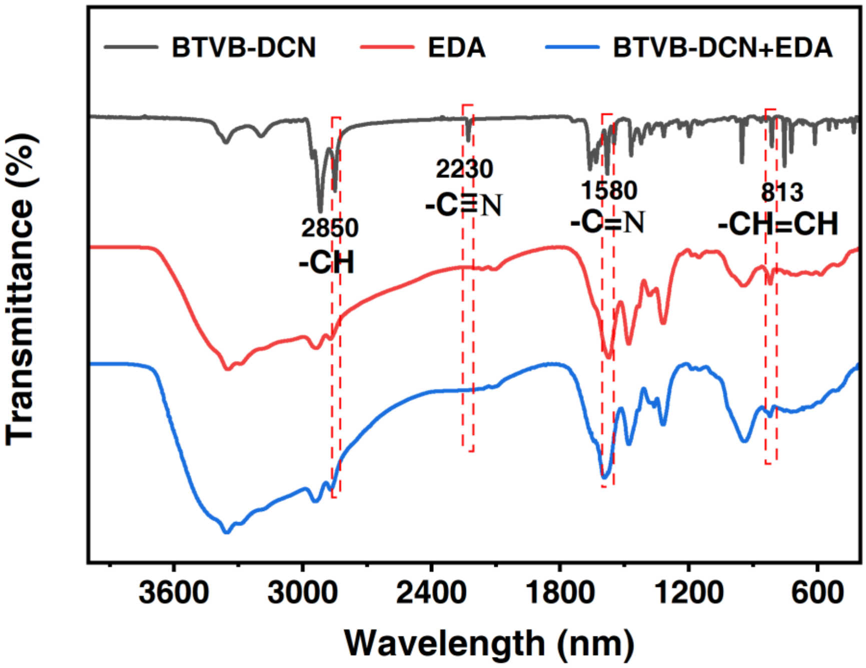

Figure S14. FT-IR spectra of the probe BTVB-DCN before and after detecting EDA.

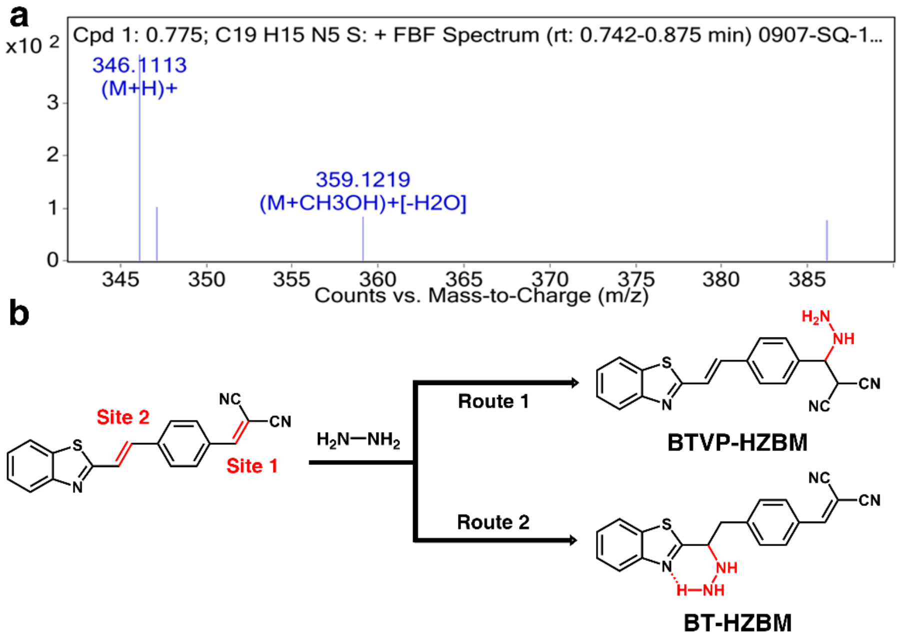

Figure S15. Hypothesized reaction products upon the recognition reaction between the probe towards hydrazine. (a) HRMS spectrum of the hypothesized products 2-((4-(2-(benzo[d]thiazol-2-yl)vinyl)phenyl)(hydrazineyl)methyl)malononitrile (BTVP-HZBM) and 2-(4-(2-(benzo[d]thiazol-2-yl)-1-hydrazineylethyl)benzylidene)malononitrile (BT-HZBM); (b) Hypothesized reaction routes and products between the probe and hydrazine.

Note: According to the peak of 346.1113 (m/z) ($M+H^+$) in HRMS spectrum (**Figure S15a**) collected after the probe interacting with hydrazine (N_2H_4), there were two potential reaction routes and corresponding products (**Figure S15b**).

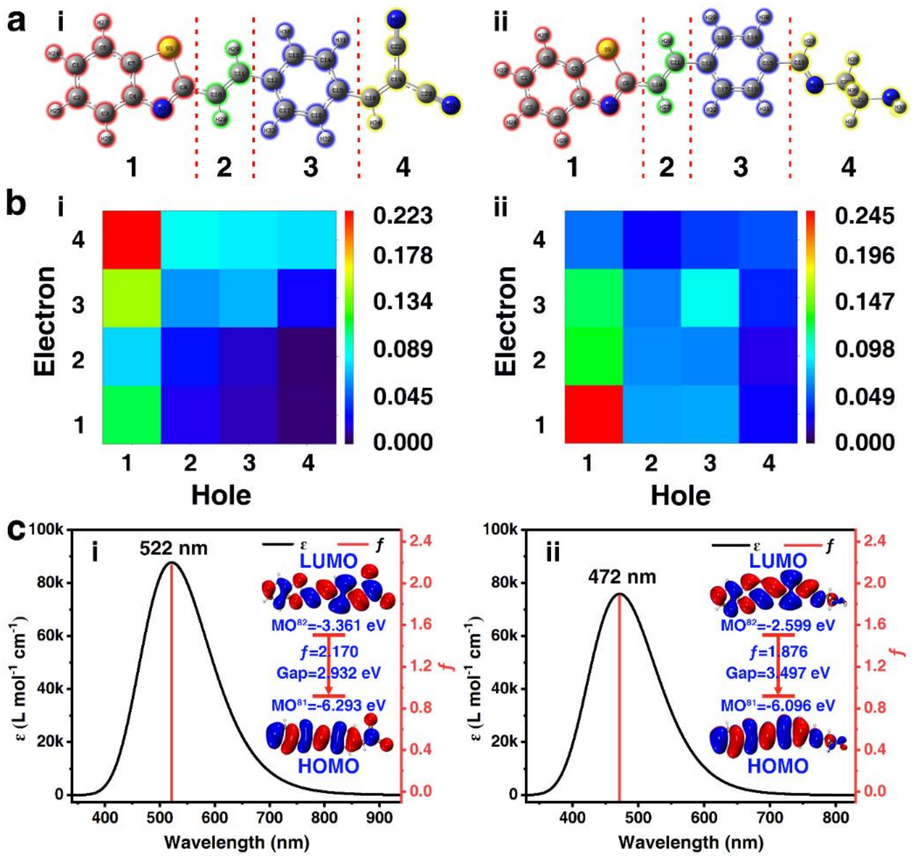

Figure S16. Theoretical computation analysis of the optical sensing mechanism of the probe BTVB-DCN towards EDA. (a) The division of the molecule structure for the hole-electron analysis, i) the BTVB-DCN probe and ii) the product BTVB-EA; (b) the fragment transition density matrix plots, i) the BTVB-DCN probe and ii) the product BTVB-EA; (c) Simulated fluorescence spectra of i) the BTVB-DCN probe and ii) the product BTVB-EA, the oscillator strength, the main contribution of the molecular orbital (MO) transition from S1→S0 emission and corresponding energy gaps were labelled, the red and blue regions indicate the positive and negative orbital phases, respectively.

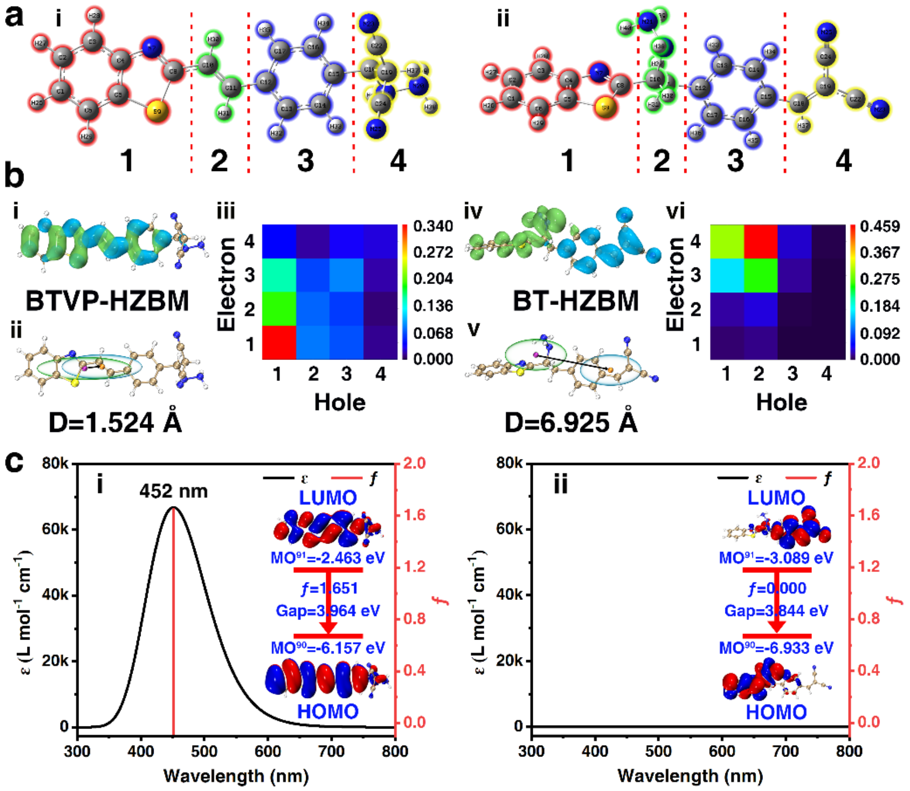

Figure S17. Theoretical computation analysis of the optical sensing mechanism of the probe BTVB-DCN towards hydrazine (N_2H_4). (a) Division of the molecule structure for the hole-electron analysis i) the product BTVP-HZBM and ii) the product BT-HZBM; (b) Hole-electron distribution analysis for the product BTVP-HZBM and the product BT-HZBM: i), iv) the hole and electron distributions, ii), v) the $C_{hole}/C_{electron}$ plots smoothly transformed from the hole and electron distributions, the centroids of the Chole and Celectron were marked by purple and orange spheres, respectively, and the charge transfer distances were labelled as D, iii), vi) the fragment transition density matrix plots; (c) Simulated fluorescence spectra of i) the product BTVP-HZBM and ii) the product BT-HZBM, the oscillator strength, the main contribution of the molecular orbital (MO) transition from S1→S0 emission and corresponding energy gaps were labelled, the red and blue regions indicate the positive and negative orbital phases, respectively.

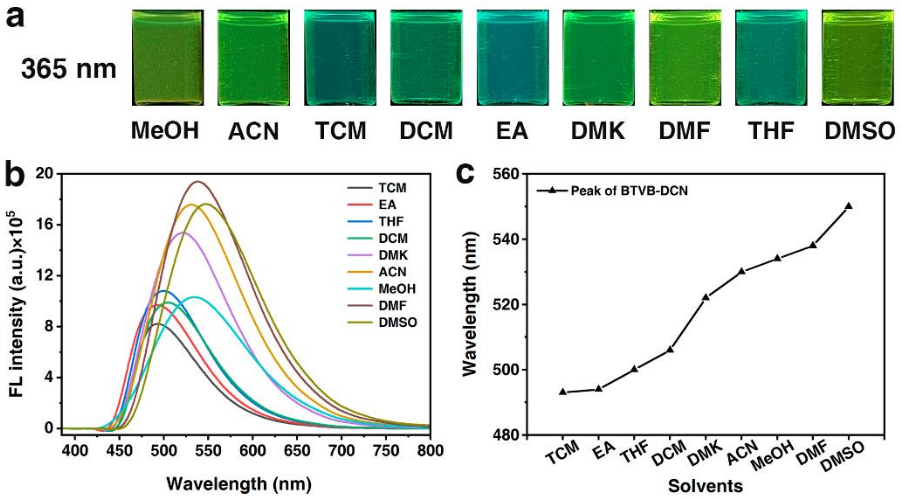

Figure S18. Optimization of solvent type of the probe: (a) optical images under 365 nm illumination; (b) fluorescence spectra, and (c) the corresponding emission intensity.

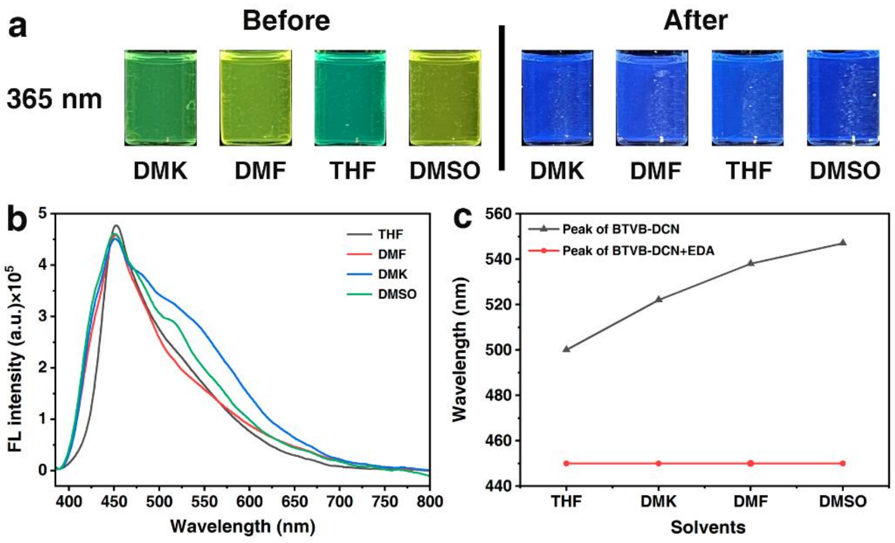

Figure S19. Optimization of solvent type of the probe upon the sensing response towards EDA: (a) optical images under 365 nm illumination; (b) fluorescence spectra, and (c) the corresponding emission intensity.

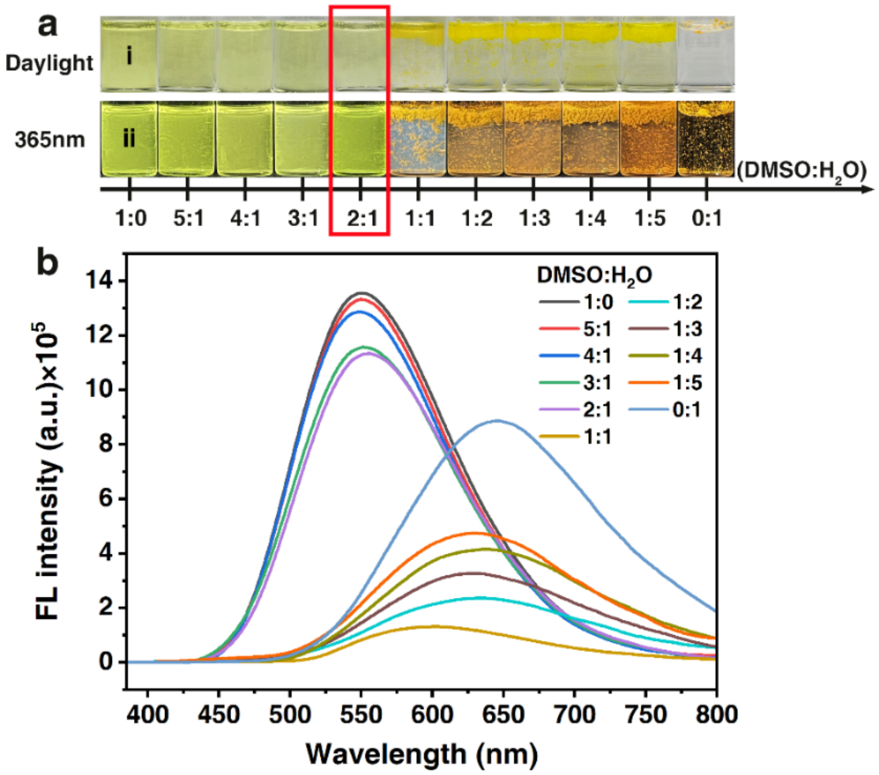

Figure S20. Optimization of the volume ratio of the mixed DMSO/H_2O for probe: (a) optical images; (b) fluorescence spectra.

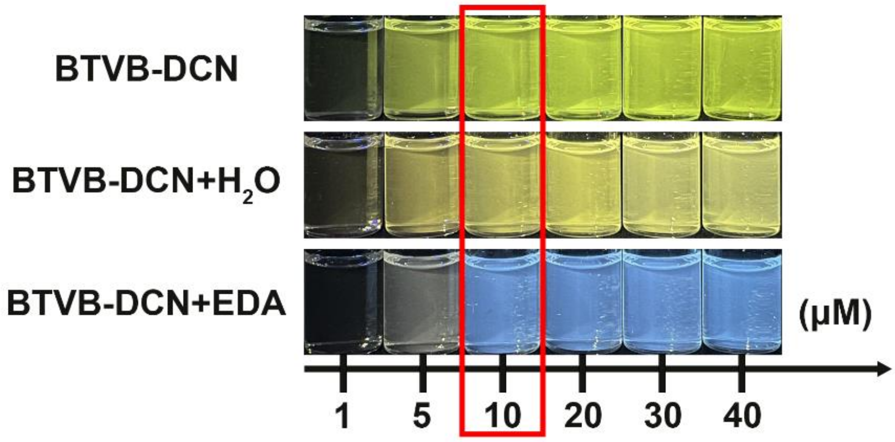

Figure S21. Fluorescence images for optimizing the probe concentration.

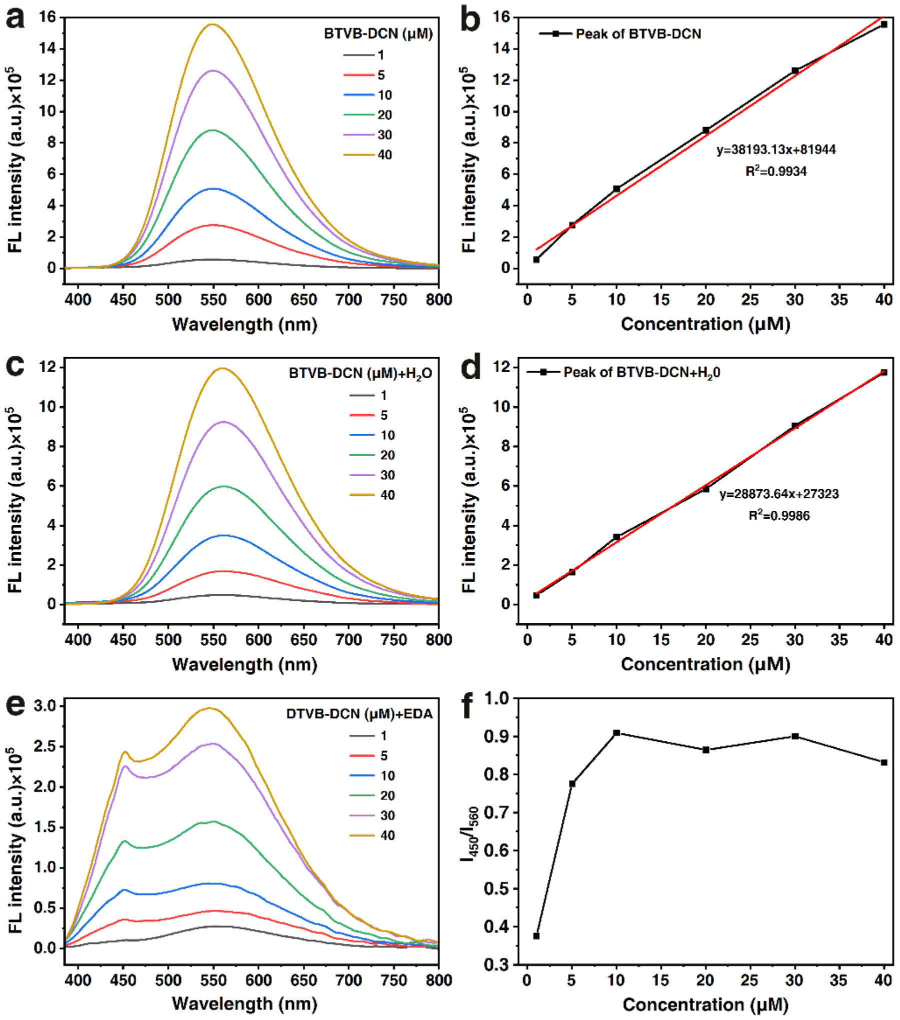

Figure S22. Fluorescence spectra and the corresponding emission intensities for optimizing the probe concentration.

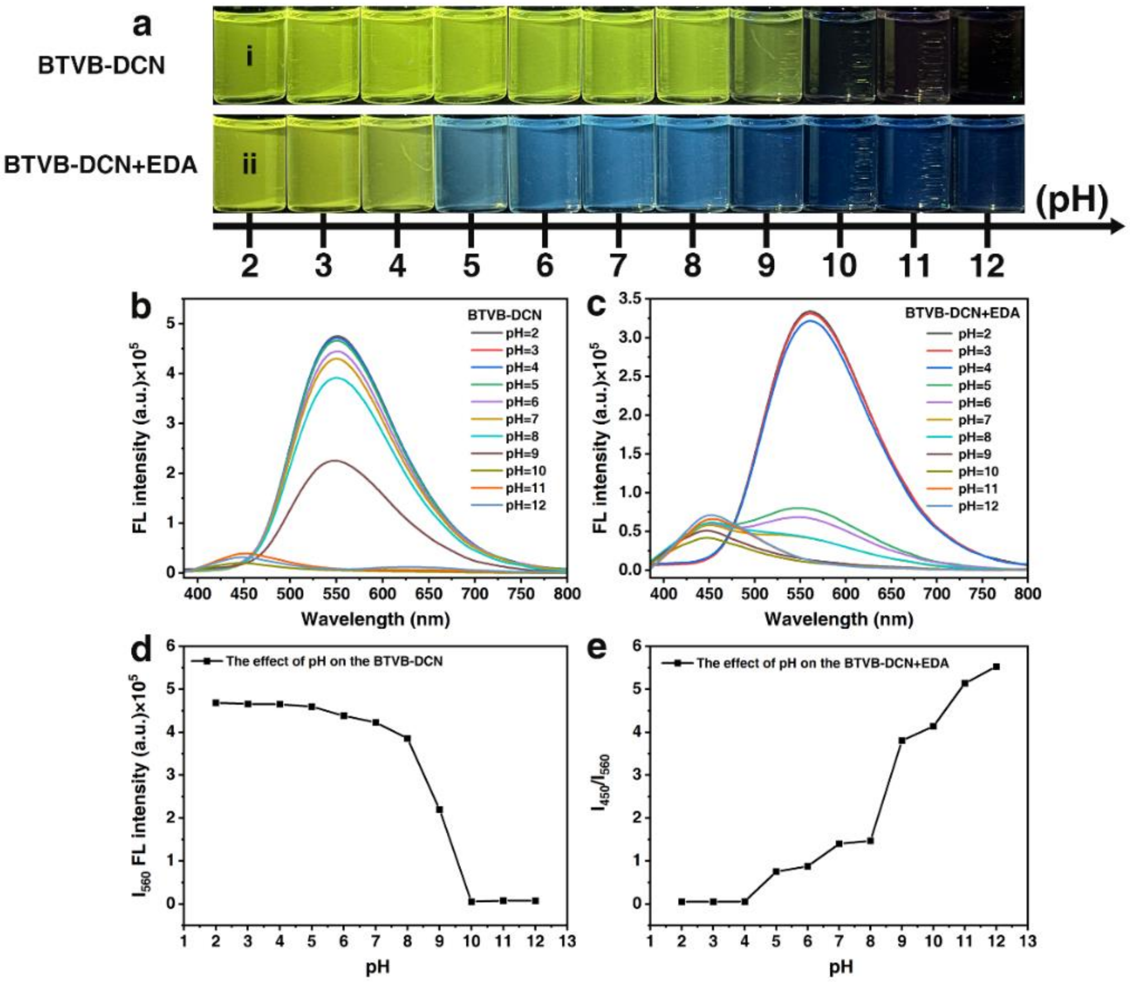

Fig. S23. Optimization of the pH of the probe before and after detecting EDA: (a) optical images, the corresponding (b-c) fluorescence spectra and (d-e) emission intensities.

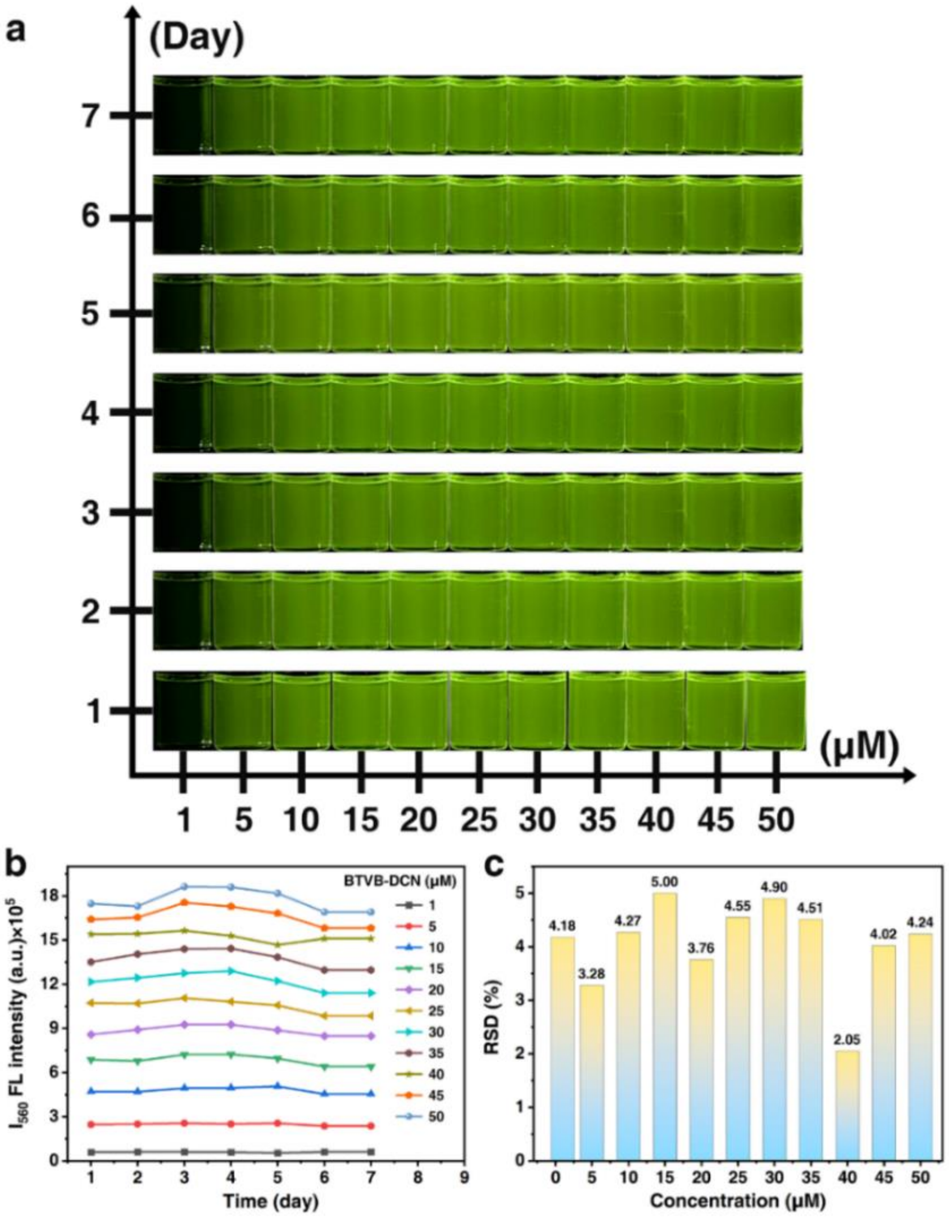

Figure S24. Stability study of the probe with different concentrations for 7 days: (a) optical images, (b) the corresponding emission intensities and (c) relative standard deviations (RSDs).

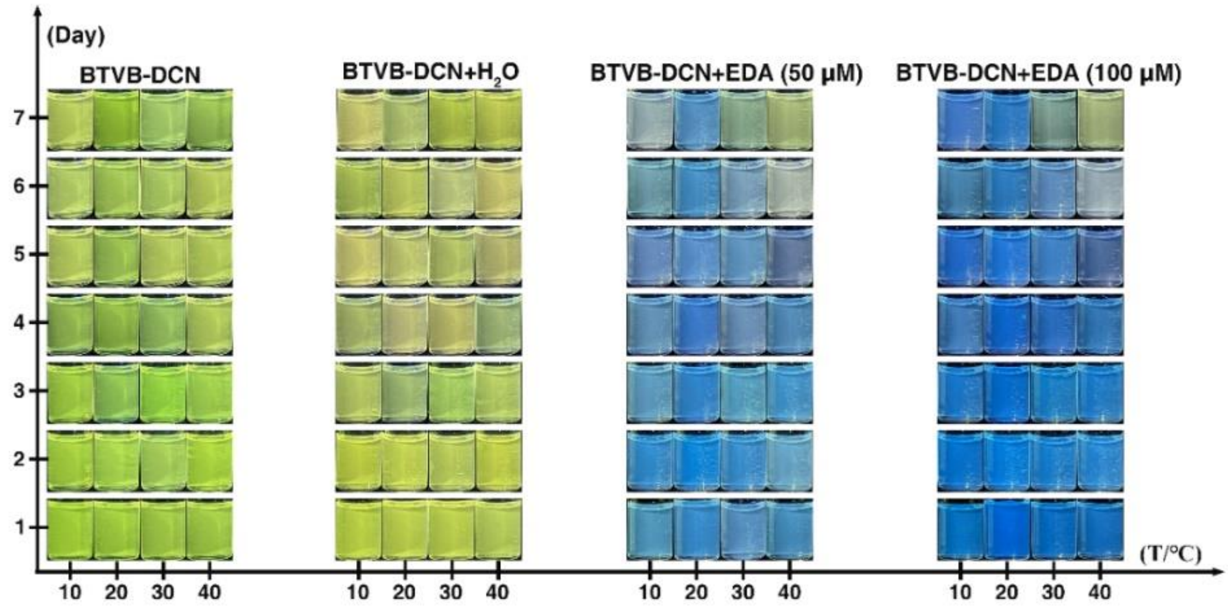

Figure S25. Optical images of stability study of the probe before and after detecting EDA under different temperatures for 7 days.

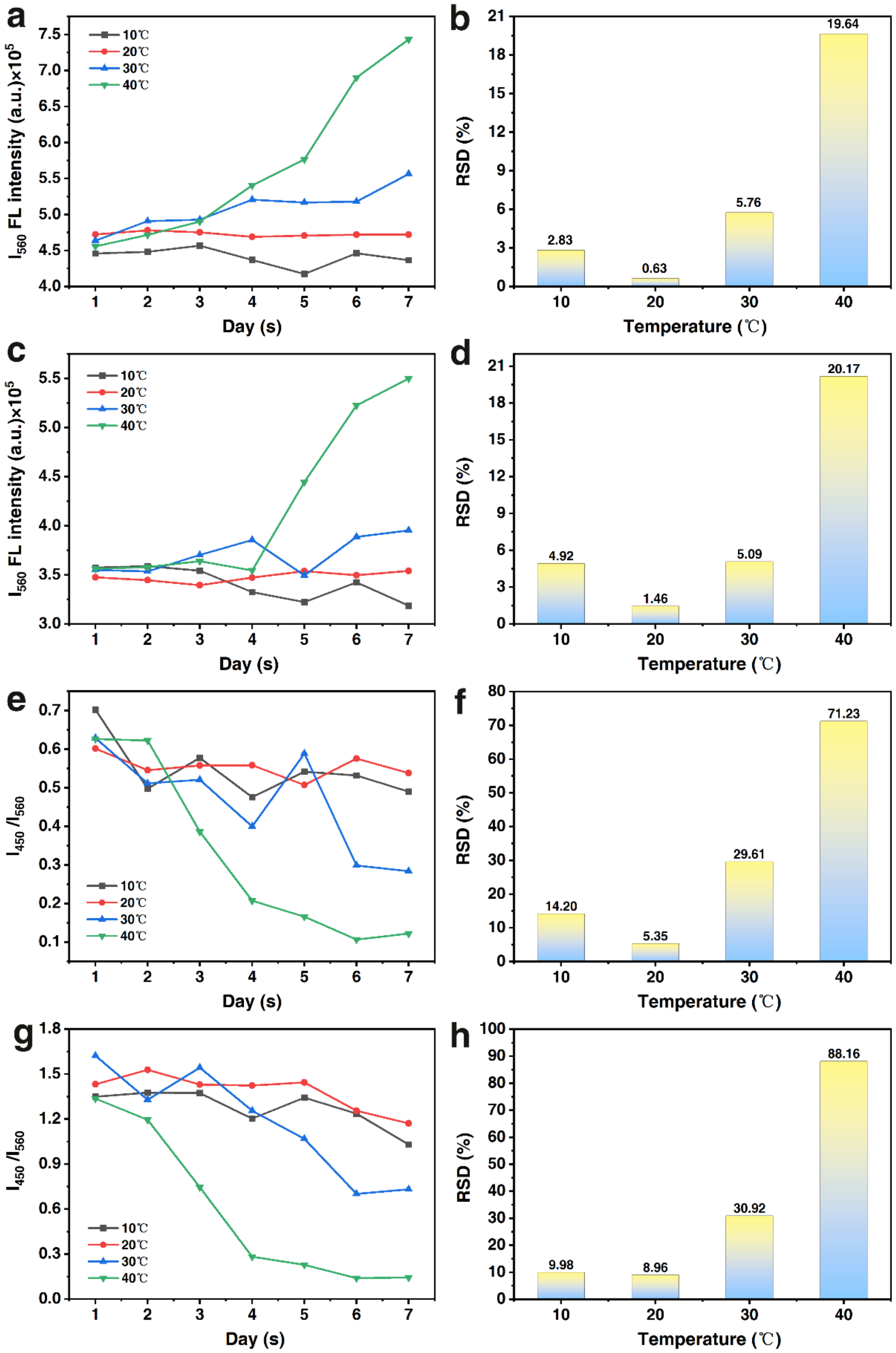

Figure S26. Stability study of the probe before and after detecting EDA under different temperatures for 7 days: (a, c, e, g) the corresponding emission intensities and intensity ratios, (b, d, f, h) relative standard deviation (RSD) for the measurements under different temperatures.

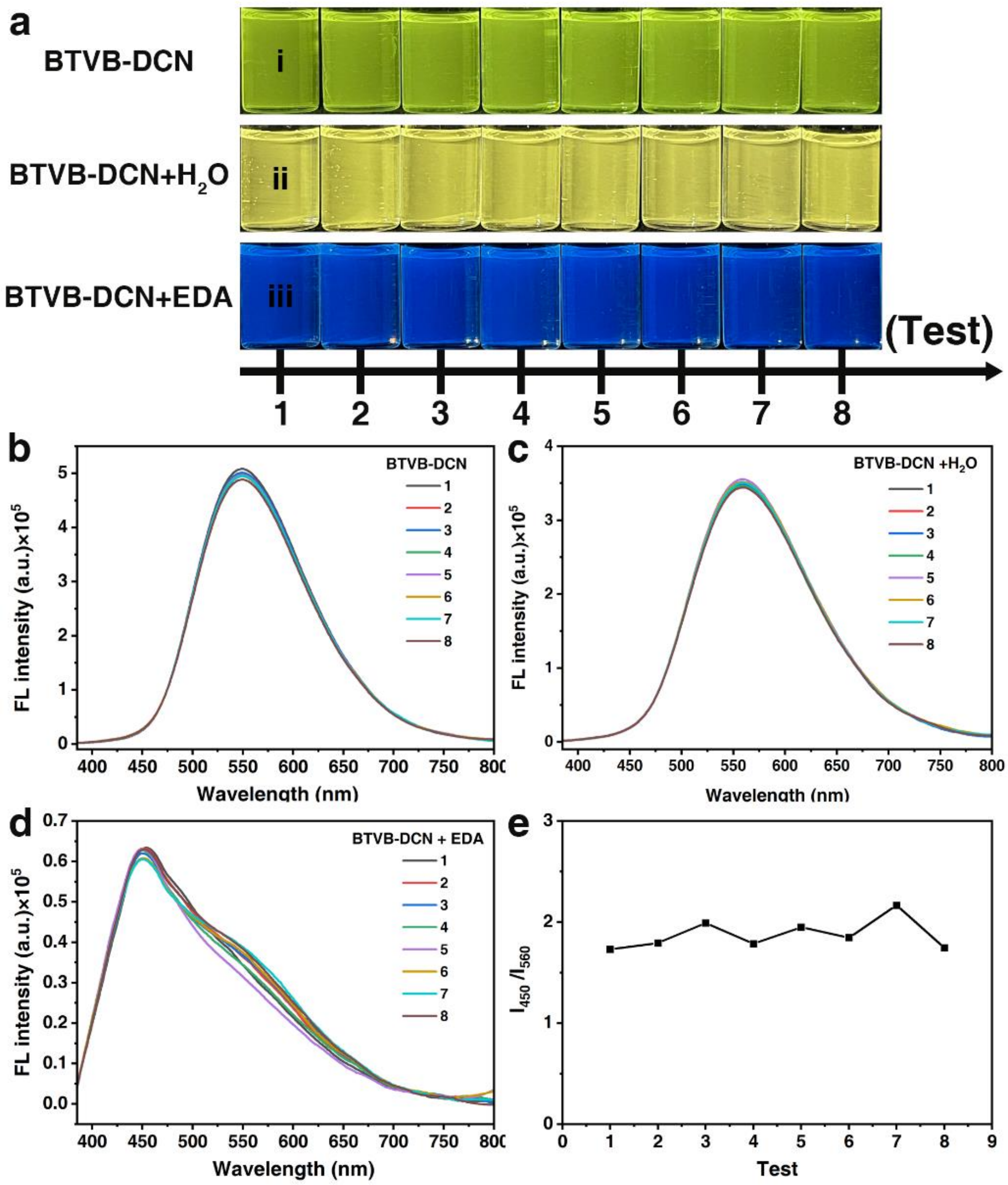

Figure S27. Repeatability study of the probe before and after detecting EDA: (a) optical images, (b-d) the corresponding images and (e) emission intensities.

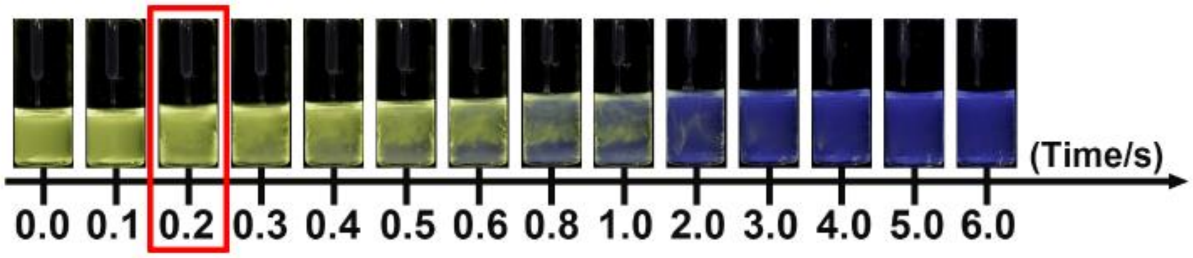

Figure S28. Evaluation of response time.

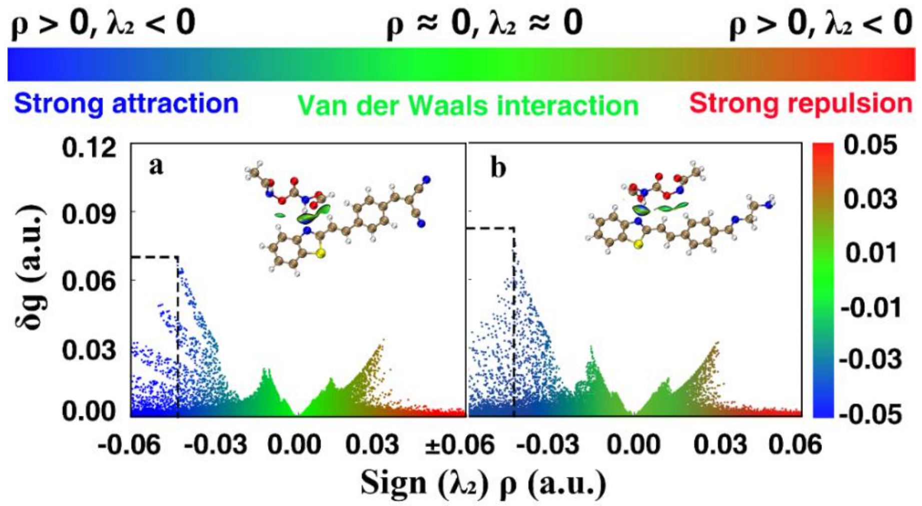

Figure S29. Scatter graph and isosurface of non-covalent interactions between the PU substrate and (a) the probe BTVB-DCN, (b) the product BTVB-EA, respectively.

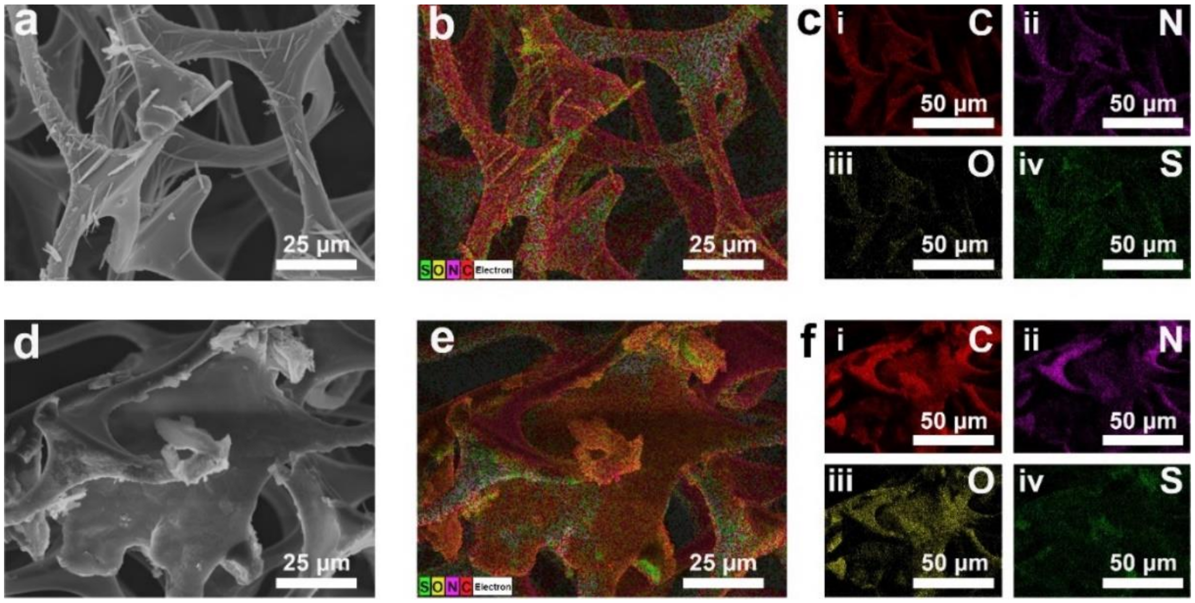

Figure S30. SEM images of the probe before (a) and after (d) interacting with EDA vapour on the PU substrate; EDS analysis of the probe before (b) and after (e) interacting with EDA vapour on the PU substrate; Distribution of the specific element for the probe before (c) and after (f) interacting with EDA vapour on the PU substrate: i) C, ii) N, iii) O, iv) S.

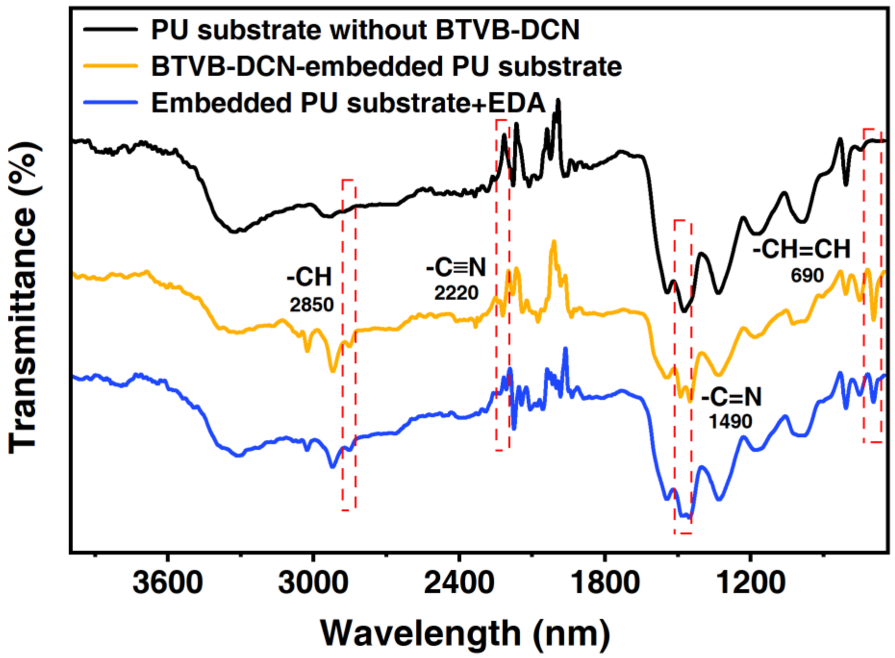

Figure S31. ATR-FTIR spectra of the probe BTVB-DCN embedded PU substrate before and after interacting with EDA.

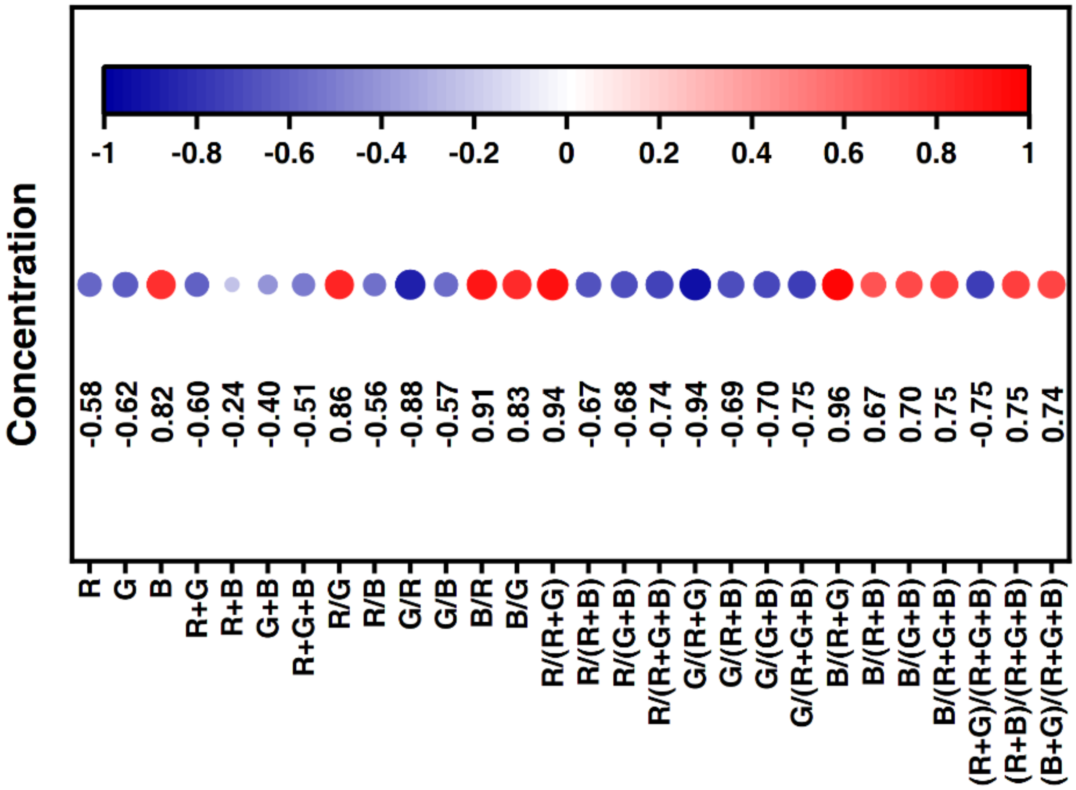

Figure S32. Correlation analysis between EDA concentration and different RGB values of the sensing substrate.

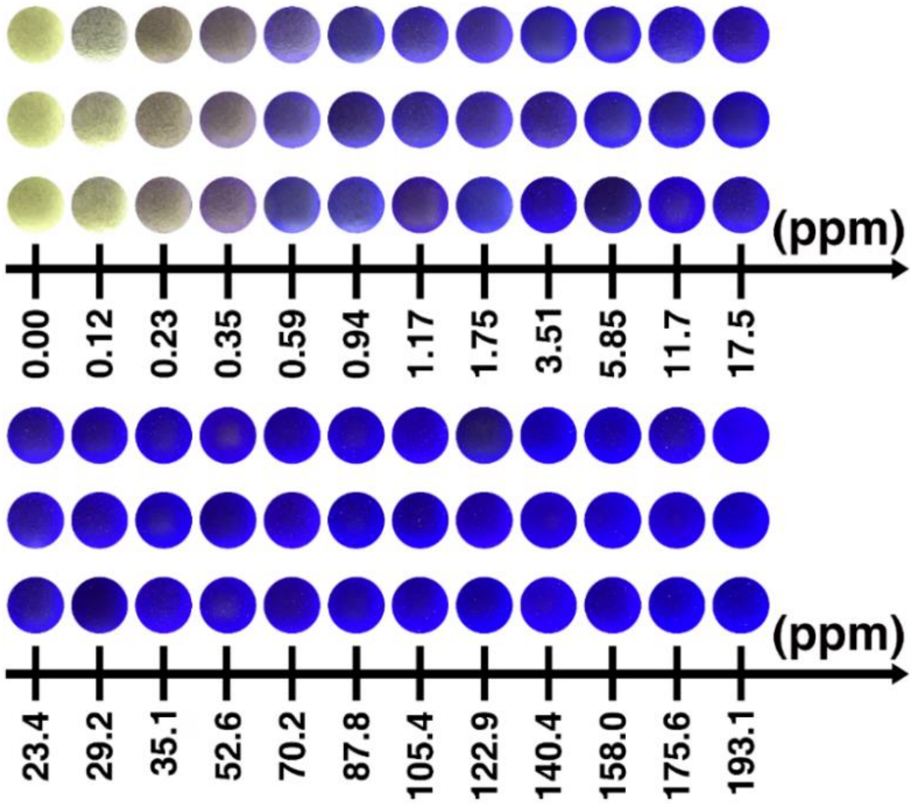

Figure S33. Optical images of the functionalized PU substrate towards EDA vapours with a series of concentrations (n=3).

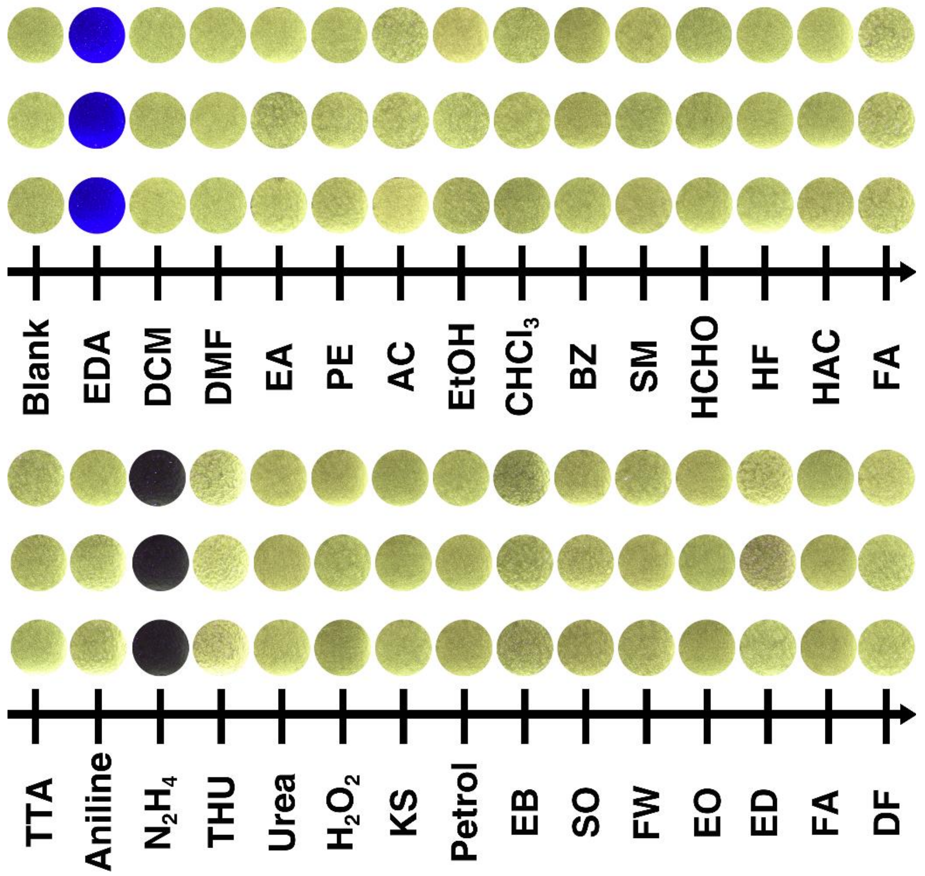

Figure S34. Optical images for specificity study of the functionalized substrate in response to EDA and other potential interferents (n=3).

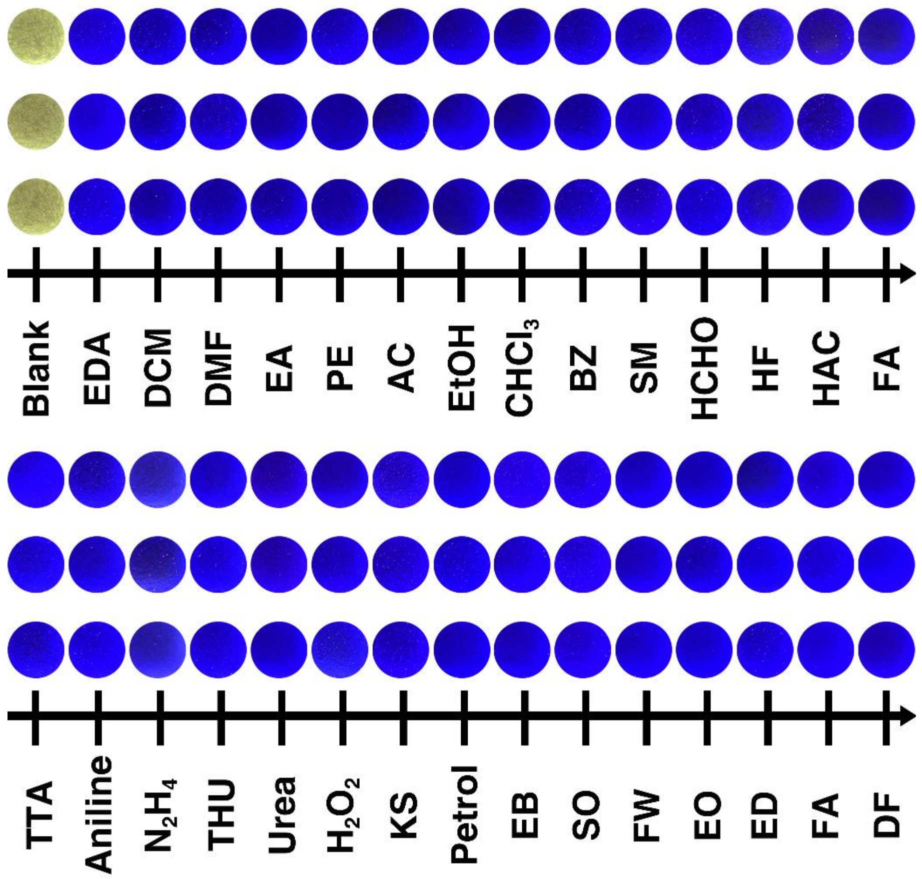

Figure S35. Optical images for anti-interferent ability of the functionalized substrate in response to the mixture of EDA and other potential interferents (n=3).

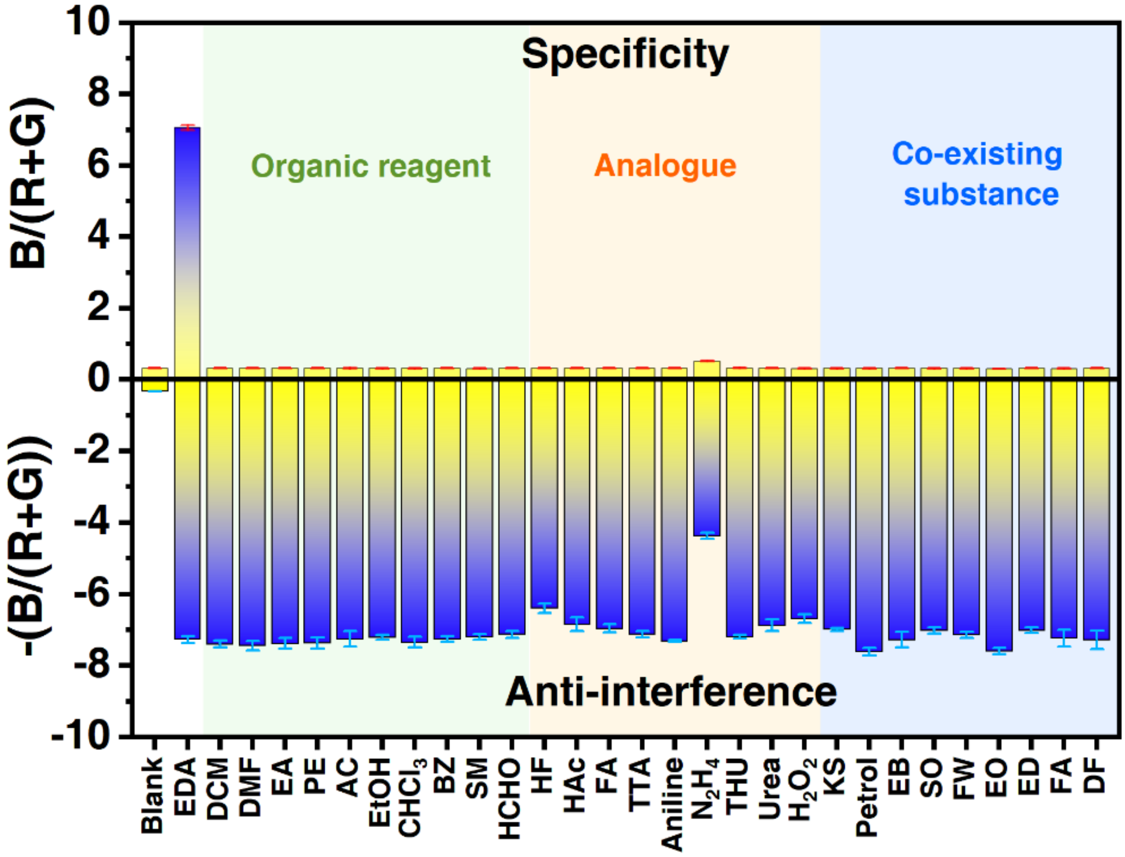

Figure S36. Histogram of the B/R+G value to each analyte in specificity and anti-interferent ability studies.

Note: The error bar represents three experimental replicates.

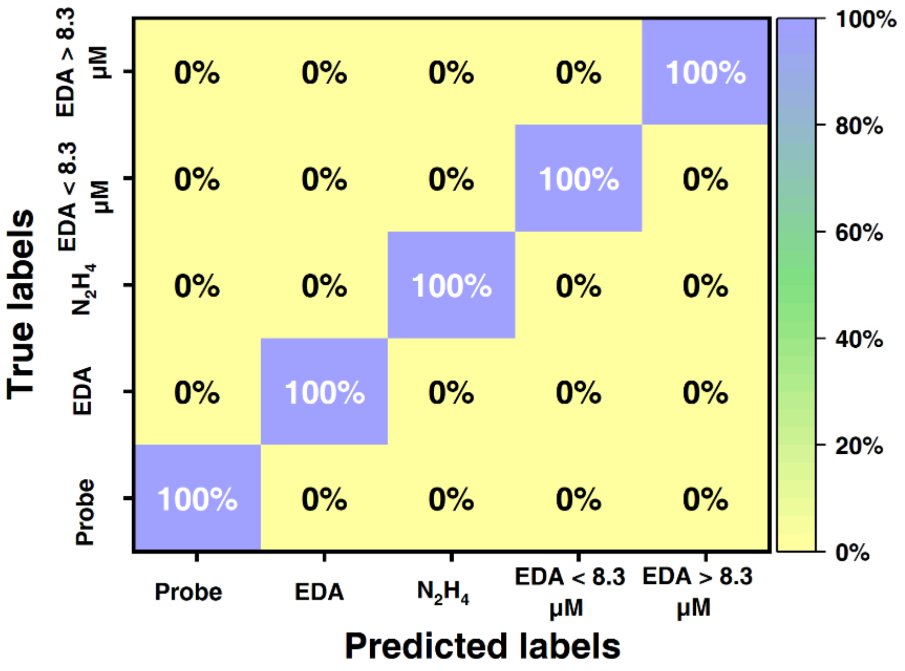

Figure S37 Judgement outcomes the randomly selected data from the test set based on the prediction model.

Note: There were 141 data in total, including 33 data for the probe, 30 data for 0.12~1.8 ppm EDA vapour, 30 data for 100 ppm N_2H_4 vapour, 30 data for 1.7~8.3 μM EDA solution (for the group of EDA<8.3 μM), 18 data for 8.3~35.1 μM EDA solution (for the group of EDA>8.3 μM).

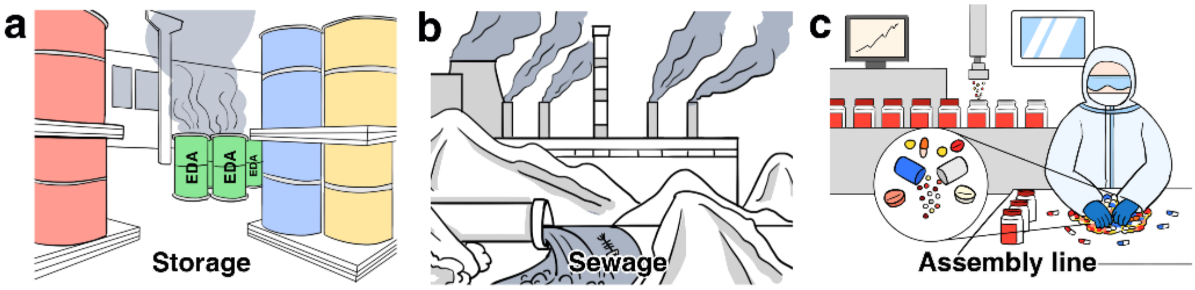

Figure. S38. Schematic diagram of the potential application scenarios, including (a) the storage garage of hazardous chemicals, (b) the discharge of industrial sewage, (c) the assembly line of medication.

Table S1. Crystal data and structure refinement of BTVB-DCN

**Compound**	**BTVB-DCN**
Empirical formula	$C_{19}H_{11}N_3S$
Formula weight	313.37
Temperature/K	293.10
Crystal system	monoclinic
Space group	$P2_1/n$
a/Å	3.8865(9)
b/Å	19.982(5)
c/Å	19.241(5)
α/°	90
β/°	91.792(9)
γ/°	90
Volume/Å^3	1493.5(6)
Z	4
ρ_{calc}g/cm^3	1.394
μ/mm^{-1}	0.219
F(000)	648.0
Crystal size/mm^3	0.13 × 0.12 × 0.1
Radiation	Mo Kα (λ = 0.71073)
2Θ range for data collection/°	4.236 to 54.962
Index ranges	$-5 \le h \le 5$, $-25 \le k \le 25$, $-20 \le l \le 24$
Reflections collected	13536
Independent reflections	3388 [R_{int} = 0.0485, R_{sigma} = 0.0477]
Data/restraints/parameters	3388/0/208
Goodness-of-fit on F^2	1.183
Final R indexes [I>=2σ (I)]	R_1= 0.0664, wR_2 = 0.1450
Final R indexes [all data]	R_1= 0.0971, wR_2 = 0.1597
Largest diff. peak/hole / e Å^{-3}	0.49/-0.21

Note: The obtained single crystal has been deposited at the Cambridge Crystallographic Data Centre and allocated the deposition number: 2269624.

Table S2. Electron-hole analysis of the probe molecule and the product

Molecule	Oscillator Strength	HOMO (eV)	LUMO (eV)	HOMO /LUMO Gap (eV)	Excitation energy (eV)	Theoratical emission wavelength (nm)	Experimental emission wavelength (nm)
BTVB-DCN	2.170	-6.293	-3.361	2.932	2.377	522	560
BTVB-EA	1.876	-6.096	-2.599	3.497	2.628	472	450
BTVP-HZDM	1.651	-6.157	-2.463	3.964	2.745	452	0
BT-HZDM	0.000	-6.933	-3.089	3.844	0.045	0	0
**Molecule**	**Sr (a.u.)**	**Sm (a.u.)**	**D (Å)**	**H (Å)**	**t (Å)**	**HDI**	**EDI**
BTVB-DCN	0.750	0.489	2.847	4.389	-1.332	5.610	5.910
BTVB-EA	0.803	0.580	1.034	3.930	-2.652	5.850	6.340
BTVP-HZDM	0.799	0.536	1.339	4.372	-2.850	6.30	6.67
BT-HZDM	0.158	0.031	6.925	2.758	4.617	14.44	8.41

Table S3. Comparison for the main performances of the reported detection methods and this work

Technique	Response time	EDA state	Linear range	LOD	Specificity	Synchronous recognition of hydrazine	Reference
Fluorometry	-	Liquid	0~50 μM	410 nM	5 kinds	-	[24]
Fluorometry	-	Liquid	0~1 mM	μM	9 kinds	-	[25]
Fluorometry	-	Vapour	-	-	12 kinds	-	[26]
Colorimetry	120 s	Vapour	-	5.6 ppm	10 kinds	-	[27]
Fluorometry	-	Liquid	0~50 μM<br>0~50 μM	1.2 μM<br>0.8 μM	8 kinds	-	[28]
Colorimetry	-	Liquid	-	-	-	-	[29]
Fluorometry	-	Liquid	-	28.3 nM	10 kinds	-	[30]
Fluorometry	75 min	Vapour	-	132 ppm	13 kinds	-	[31]
Colorimetry	10 min	Vapour	-	-	-	-	[32]
Fluorometry	-	Liquid	0~1 mM	0.87 μM	12 kinds	-	[33]
Fluorometry<br>Colorimetry	< 4 s<br>90 s	Liquid<br>Vapour	0.5~80 μM (Colorimetry)<br>0.5~10 μM (Fluorometry)<br>3.2~48 ppm (Colorimetry)<br>3.2~64 ppm (Fluorometry)	42 nM (Colorimetry)<br>0.17 nM (Fluorometry)<br>3.2 ppm (Colorimetry)<br>3.2 ppm (Fluorometry)	28 kinds<br>17 kinds	Cannot recognize hydrazine	[34]
Fluorometry	-	Liquid<br>Vapour	No	54 nM<br>-	22 kinds<br>-	-<br>-	[35]
**Fluorometry**	**0.2 s**<br>**90 s**	**Liquid**<br>**Vapour**	**0~33.3 μM**<br>**0~193 ppm**	**8.6 nM**<br>**1.61 ppb**	**32 kinds***<br>**28 kinds**	**Yes**<br>**Yes**	**This work**

Note: "-" stands for "Did not mention".

"*": 32 kinds of potential interferents were separately studied in Figure S11 of section "Theoretical computation analysis of optical sensing mechanism" and Figure 2 of section "Fluorescent sensing performances of the BTVB-DCN Probe to EDA solution".

Reference

[1] Y. L. Pan, Z. B. Cai, L. Bai, F. F. Ma, S. L. Li, Y. P. Tian, *Tetrahedron* **2017**, *73*, 2886.
[2] D. X. Cao, Z. Q. Liu, G. H. Zhang, F. X. Cao, H. Y. Chen, G. Z. Li, *Dyes Pigment.* **2008**, *76*, 118.
[3] H. L. Zhao, J. H. Wu, X. J. Meng, S. H. Zuo, W. T. Wang, H. H. Yuan, M. B. Lan, *J. Heterocycl. Chem.* **2008**, *45*, 371.
[4] O. V. Dolomanov, L. J. Bourhis, R. J. Gildea, J. A. K. Howard, H. Puschmann, *J. Appl. Crystallogr.* **2009**, *42*, 339.
[5] G. M. Sheldrick, *Acta Crystallogr. Sect. A* **2015**, *71*, 3.
[6] G. M. Sheldrick, *Acta Crystallogr. Sect. C Struct. Chem.* **2015**, *71*, 3.
[7] M. J. Frisch, G. W. Trucks, H. B. Schlegel, G. E. Scuseria, M. A. Robb, J. R. Cheeseman, G. Scalmani, V. Barone, G. A. Petersson, H. Nakatsuji, X. Li, M. Caricato, A. V. Marenich, J. Bloino, B. G. Janesko, R. Gomperts, B. Mennucci, H. P. Hratchian, J. V. Ortiz, A. F. Izmaylov, J. L. Sonnenberg, F. Williams Ding, F. Lipparini, F. Egidi, J. Goings, B. Peng, A. Petrone, T. Henderson, D. Ranasinghe, V. G. Zakrzewski, J. Gao, N. Rega, G. Zheng, W. Liang, M. Hada, M. Ehara, K. Toyota, R. Fukuda, J. Hasegawa, M. Ishida, T. Nakajima, Y. Honda, O. Kitao, H. Nakai, T. Vreven, K. Throssell, J. A. Montgomery Jr., J. E. Peralta, F. Ogliaro, M. J. Bearpark, J. J. Heyd, E. N. Brothers, K. N. Kudin, V. N. Staroverov, T. A. Keith, R. Kobayashi, J. Normand, K. Raghavachari, A. P. Rendell, J. C. Burant, S. S. Iyengar, J. Tomasi, M. Cossi, J. M. Millam, M. Klene, C. Adamo, R. Cammi, J. W. Ochterski, R. L. Martin, K. Morokuma, O. Farkas, J. B. Foresman, D. J. Fox, In *Gaussian 09 Rev. A.02*, Wallingford, CT, **2009**.
[8] S. Grimme, S. Ehrlich, L. Goerigk, *J. Comput. Chem.* **2011**, *32*, 1456.
[9] A. Kenane, D. Hadji, K. Argoub, A. Yahiaoui, A. Hachemaoui, K. Toubal, A. M. Benkouider, O. Rasoga, A. Stanculescu, A. C. Galca, *J. Electron. Mater.* **2023**, *52*, 530.
[10] F. Weigend, *Phys. Chem. Chem. Phys.* **2006**, *8*, 1057.
[11] F. Weigend, R. Ahlrichs, *Phys. Chem. Chem. Phys.* **2005**, *7*, 3297.
[12] G. Scalmani, M. J. Frisch, B. Mennucci, J. Tomasi, R. Cammi, V. Barone, *J. Chem. Phys.* **2006**, *124*.
[13] S. Grimme, *Wiley Interdiscip. Rev. Comput. Mol. Sci.* **2011**, *1*, 211.
[14] J. L. Pascual-ahuir, E. Silla, I. Tuñon, *J. Comput. Chem.* **1994**, *15*, 1127.
[15] S. Miertuš, E. Scrocco, J. Tomasi, *Chem. Phys.* **1981**, *55*, 117.
[16] A. Gabrieli, M. Sant, P. Demontis, G. B. Suffritti, *J. Chem. Theory Comput.* **2015**, *11*, 3829.

[17] C. Lefebvre, G. Rubez, H. Khartabil, J. C. Boisson, J. Contreras-García, E. Hénon, *Phys. Chem. Chem. Phys.* **2017**, *19*, 17928.

[18] Z. Y. Liu, T. Lu, Q. X. Chen, *Carbon* **2020**, *165*, 461.

[19] T. Lu, F. W. Chen, *J. Comput. Chem.* **2012**, *33*, 580.

[20] W. Humphrey, A. Dalke, K. Schulten, *J. Mol. Graph.* **1996**, *14*, 33.

[21] T. Lu, Q. X. Chen, *Comput. Theor. Chem.* **2021**, *1200*, 113249.

[22] Y. Lei, Q. Chen, P. R. Liu, L. X. Wang, H. Y. Wang, B. D. Li, X. Y. Lu, Z. Chen, Y. J. Pan, F. H. Huang, H. Li, *Angew. Chem. Int. Ed.* **2021**, *60*, 4705.

[23] W. Y. Guan, S. O. Santana, J. N. Liao, K. Henninger, M. P. Watson, *ACS Catal.* **2020**, *10*, 13820.

[24] Y. Kim, S. H. Son, T. S. Lee, *Mol. Cryst. Liquid Cryst.* **2014**, *600*, 179.

[25] Y. M. Xu, S. S. Yu, Y. C. Wang, L. L. Hu, F. Zhao, X. M. Chen, Y. N. Li, X. Q. Yu, L. Pu, *Eur. J. Org. Chem.* **2016**, *2016*, 5868.

[26] P. Li, D. Q. Yang, H. R. Li, *Dyes Pigment.* **2016**, *132*, 306.

[27] Y. J. Jin, G. Kwak, *Sens. Actuators B Chem.* **2018**, *271*, 183.

[28] S. B. Seenivasagaperumal, S. Shanmugam, *New J. Chem.* **2018**, *42*, 3394.

[29] J. B. Essner, G. A. Baker, *Anal. Bioanal. Chem.* **2018**, *410*, 4607.

[30] M. Saravanakumar, B. Umamahesh, R. Selvakumar, J. Dhanapal, S. K. A. Kumar, K. I. Sathiyanarayanan, *Dyes Pigment.* **2020**, *178*, 108346.

[31] S. Tunsrichon, C. Sukpattanacharoen, D. Escudero, N. Kungwan, S. Youngme, J. Boonmak, *Inorg. Chem.* **2020**, *59*, 6176.

[32] J. J. Liu, Z. L. Sun, J. M. Liu, S. B. Xia, *J. Mol. Struct.* **2021**, *1238*, 130444.

[33] P. M. Chuang, Y. J. Tu, J. Y. Wu, *Sens. Actuators B Chem.* **2022**, *366*.

[34] Y. L. Ke, Y. Liu, B. Y. Zu, D. Lei, G. F. Wang, J. G. Li, W. F. Ren, X. C. Dou, *Angew. Chem. Int. Ed.* **2022**, *61*, e202203358.

[35] B. Priya, N. Kumar, Roopa, *J. Photochem. Photobiol. A Chem.* **2023**, *445*, 115036.
